# Molecular Phylogeny of Unicellular Marine Coccoid Green Algae Revealed New Insights into the Systematics of the Ulvophyceae (Chlorophyta)

**DOI:** 10.3390/microorganisms9081586

**Published:** 2021-07-26

**Authors:** Tatyana Darienko, Cecilia Rad-Menéndez, Christine N. Campbell, Thomas Pröschold

**Affiliations:** 1 Albrecht-von-Haller-Institute of Plant Sciences, Experimental Phycology and Culture Collection of Algae, Georg-August-University of Goettingen, D-37073 Göttingen, Germany; tdarien@gwdg.de; 2 Research Department for Limnology, Leopold-Franzens-University of Innsbruck, A-5310 Mondsee, Austria; 3 Collection of Algae and Protozoa, Scottish Association for Marine Science, Oban PA37 1QA, UK; Cecilia.RadMenendez@sams.ac.uk (C.R.-M.); Christine.Campbell@sams.ac.uk (C.N.C.)

**Keywords:** *Desmochloris*, *Chlorocystis*, *Halochlorococcum*, *Pseudoneochloris*, *Sykidion*, molecular phylogeny, small subunit rRNA (SSU), internal transcribed spacer (ITS)

## Abstract

Most marine coccoid and sarcinoid green algal species have traditionally been placed within genera dominated by species from freshwater or soil habitats. For example, the genera *Chlorocystis* and *Halochlorococcum* contain exclusively marine species; however, their familial and ordinal affinities are unclear. They are characterized by a vegetative cell with lobated or reticulated chloroplast, formation of quadriflagellated zoospores and living epi- or endophytically within benthic macroalgae. They were integrated into the family Chlorochytriaceae which embraces all coccoid green algae with epi- or endophytic life phases. Later, they were excluded from the family of Chlorococcales based on studies of their life histories in culture, and transferred to their newly described order, Chlorocystidales of the Ulvophyceae. Both genera form a “*Codiolum*”-stage that serves as the unicellular sporophyte in their life cycles. Phylogenetic analyses of SSU and ITS rDNA sequences confirmed that these coccoid taxa belong to the Chlorocystidales, together with the sarcinoid genus *Desmochloris*. The biflagellated coccoid strains were members of the genus *Sykidion*, which represented its own order, Sykidiales, among the Ulvophyceae. Considering these results and the usage of the ITS-2/CBC approach revealed three species of *Desmochloris*, six of *Chlorocystis*, and three of *Sykidion*. Three new species and several new combinations were proposed.

## 1. Introduction

Successful invasion of the land by marine organisms, or of the sea by freshwater organisms, is generally considered as a relatively rare event in evolution. Among the coccoid green algae, once placed together in the order Chlorococcales, there are a number of marine forms amongst the overwhelming freshwater majority. What does this represent?

Phylogenetic analyses of SSU rDNA sequences (encoding for the small subunit of the nuclear ribosomal operon) have shown that coccoid green algae, lacking flagella on their vegetative cells, are distributed among all classes of Chlorophyta (see reviews [1,2]). Most marine coccoid green algal species have traditionally been placed within genera dominated by species from freshwater or soil habitats [3]. For example, the type species of *Chlorella* (*C. vulgaris* Beijerinck) is positioned within the Trebouxiophyceae, but the marine species described as *Halochlorella rubescens* Dangeard (authentic strain SAG 5.95) is a member of the Chlorophyceae [4] and was transferred to *Scenedesmus* (*S. rubescens*) by Kessler et al. [5]. Darienko et al. [6] revealed the polyphyletic origin of marine species assigned as *Chlorella*. Some investigated strains belonged to the genus *Chloroidium* Nadson [7] and others were transferred to the newly described genus *Droopiella* Darienko et al. for those belonging to the *Oocystis* clade of the Trebouxiophyceae. Interestingly, some marine isolates were identical in sequence with the freshwater species *Chlorella vulgaris*, however, they showed different morphology under marine conditions. Only cultivated under freshwater conditions did these strains reveal the typical *C. vulgaris* morphology [6]. As demonstrated above, most autospore-producing taxa belonged to different lineages of the Trebouxiophyceae or Chlorophyceae. The phylogenetic position of zoospore-forming genera and species mostly remains unresolved.

Species of the genus *Neochloris* Starr are present in all three classes of the Chlorophyta (Chlorophyceae, Trebouxiophyceae, and Ulvophyceae [8]). The only marine isolate designated as *Neochloris* is a member of the Ulvophyceae (*sensu* Mattox & Stewart [9]) based on ultrastructural and phylogenetic analyses, and was described as *Pseudoneochloris marina* by Watanabe et al. [10]. This species produced biflagellated zoospores similar to those described for species of the genus *Sykidion,* Wright [11,12]. Another genus producing biflagellated zoospores is the genus *Desmochloris,* Watanabe, Kuroda & Maiwa. The type species, *D. halophila*, was originally described as *Chlorosarcinopsis halophila* by Guillard et al. [13]. Watanabe et al. [14] demonstrated that this species is a member of the Ulvophyceae based on ultrastructural and phylogenetic data. Despite this marine species, Darienko et al. [15] found a second species, *D. mollenhaueri*, isolated from different soil crusts collected in South Africa.

The genera *Chlorocystis* Reinhard and *Halochlorococcum* Dangeard ex Guiry contain exclusively marine species; however, their familial and ordinal affinities are unclear. They are characterized by a vegetative cell with lobated or reticulated chloroplast, formation of quadriflagellated zoospores, and living epi- or endophytically within benthic macroalgae. Komárek & Fott [3] integrated them into the family Chlorochytriaceae, which embraces all coccoid green algae with epi- or endophytic life phases. Kornmann & Sahling [16] excluded both these genera from this family of the Chlorococcales based on studies of their life histories in culture, and transferred them to their newly described order Chlorocystidales (Codiolophyceae *sensu* Kornmann [17]). They found, for both genera, that a “*Codiolum*”-stage served as the unicellular sporophyte in their life cycles. A “*Codiolum*”-stage was also found in marine macroalgae (*Acrosiphonia* Agardh [18,19], *Urospora* Areschoug [20,21,22], *Spongomorpha* Kützing [23,24], *Monostroma* Thuret [25], *Gomontia* Bornet & Flahault [26]) and freshwater organisms (*Ulothrix* Kützing, [27]). Kornmann [17,28] concluded that the presence of this unicellular sporophyte type in a life cycle is characteristic for these green algae and can be used for taxonomical classification. Therefore, he described a new class, Codiolophyceae (without a formal diagnosis according to the ICBN), that includes the orders Codiolales, Acrosiphonales, Monostromales, Ulotrichales, and Chlorocystidales [16,17]. O’Kelly & Floyd [29] included *Chlorocystis* and *Halochlorococcum* (“*Chlorocystis* group”) in the order Ulotrichales, within the class Ulvophyceae, which was established by Mattox & Stewart [9] based on ultrastructural features (flagellar apparatus with basal bodies in counterclockwise orientation). This class included the orders Ulotrichales, Ulvales, Siphonocladales (Cladophorales), Dasycladales, and Caulerpales [29]. However, phylogenetic analyses of partial [30] and complete [14] sequences of SSU rDNA have shown that the Ulvophyceae *sensu* Mattox and Stewart is divided into two major lineages. The first includes the orders Ulvales, Ulotrichales, and Acrosiphoniales, and the second, the Cladophorales, Dasycladales, Caulerpales, Bryopsidales, and Trentepohliales. This confusing nomenclature history needs reexaminations in light of both, DNA sequence data and the recognition of “*Codiolum*” stages. Only then can any evaluation of the evolution of lifestyle proceed. In addition to these orders mentioned above, Watanabe & Nakayama [31] demonstrated that the terrestrial sarcinoid genus *Ignatius* Bold & MacEntee, with its type species *I. tetrasporus,* belonged to the Ulvophyceae based on the ultrastructure of the quadriflagellated zoospore and phylogenetic analyses of SSU rDNA sequences. Surprisingly, almost identical in sequence was *Pseudocharacium americanum* Lee & Bold, a species occurring as epiphytic on freshwater filamentous algae. This clade of the Ulvophyceae was described as order Ignatiales by Skaloud & Leliaert in Skaloud et al. [32]. Gong et al. [33] found, in marine habitat on bleached corals, a coccoid green alga, which exclusively reproduced by aplanospores, and described it as *Symbiochlorum hainanensis*. The phylogenetic analyses revealed that this species is sister to the Ignatiales.

The aim of this study was to clarify the phylogenetic position of marine coccoid green algae, which produce bi- or quadriflagellated zoospores. An integrative approach (combination of the morphological data with molecular phylogenetic results including genetic synapomorphies) revealed that all investigated strains belong to different lineages of the Ulvophyceae. Considering these results, and in combination with detailed investigations of morphology and reproduction, several emendations among the orders and genera of the Ulvophyceae were necessary, which included the proposal of the new order Sykidiales.

## 2. Materials and Methods

### 2.1. Cultures and Light Microscopy

The strains were obtained from the Culture Collection of Algae, University of Göttingen, Germany (SAG; http://sagdb.uni-goettingen.de), the Culture Collection of Algae, University of Texas at Austin, USA (UTEX; http://www.utex.org), the Culture Collection of Algae and Protozoa, Oban, Scotland (CCAP; https://www.ccap.ac.uk), the Provasoli-Guillard National Center for Culture of Marine Phytoplankton, Bigelow, USA (NCMA formerly CCMP; https://ncma.bigelow.org), the Norwegian Culture Collection of Algae University of Oslo, Norway (NORCCA; https://niva-cca.no), and the personal collection of John A. West (now deposited at CCAP), which are listed in Table 1. The algae were grown in modified Bold Basal Medium (3N-BBM+V, medium 26a in Schlösser [34]), Seawater Medium (SWES, medium 5 in Schlösser [35]), and modified Artificial Seawater Medium (MASM, medium 25a in Schlösser [34]; added 30 mL soil extract per liter). The cultures of all species were grown in small petri dishes or in test tubes with 10 mL agar medium (1.5% *w*/*v*) at 20 °C at a photon fluence rate of 50 µE/m^2^s, and a light:dark cycle of 14:10 h. After four weeks of culture, mature vegetative cells (near the end of the light period) were observed by light microscopy and compared with original descriptions of the species (see Results and Discussion). Micrographs were made using an Olympus BX-60 microscope (Olympus, Tokyo, Japan) equipped with a Prog Res C14 plus camera and the Prog Res Capture Pro imaging system (version 2.9.0.1), both from Jenoptik, Jena, Germany.

### 2.2. DNA Extraction, PCR, Sequencing and Phylogenetic Analyses

**Figure 1:**
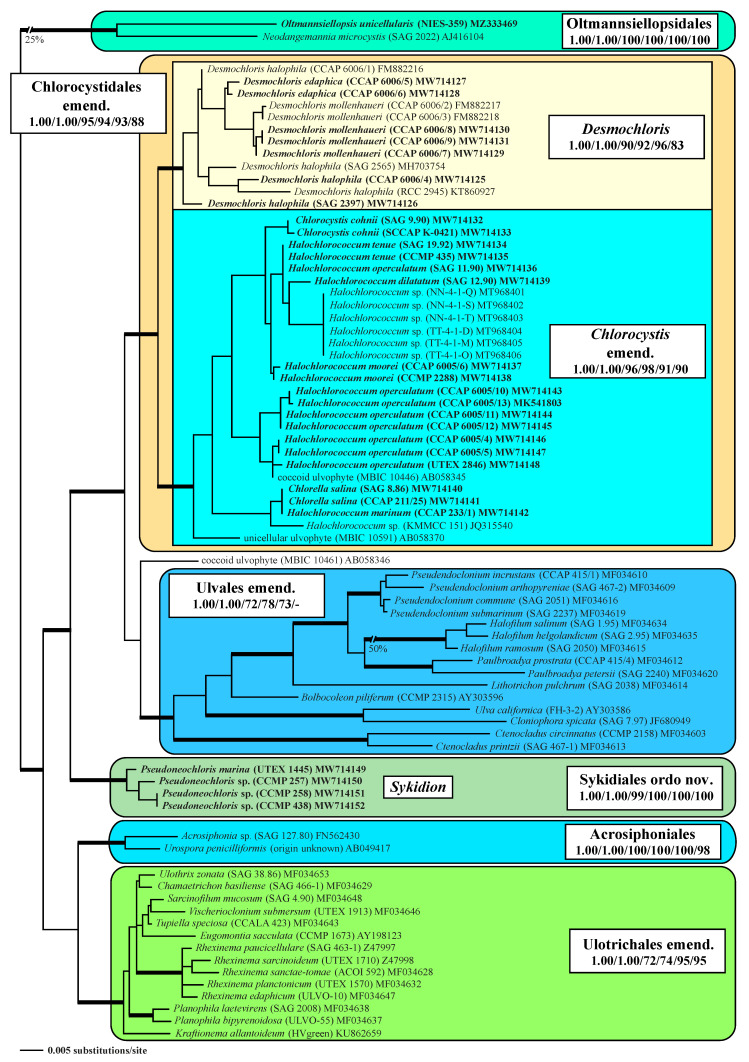
Molecular phylogeny of the Ulvophyceae based on SSU rDNA sequence comparisons. The phylogenetic tree shown was inferred using the maximum likelihood method based on a data set of 1775 aligned positions of 77 taxa using PAUP 4.0a build169. For the analysis, the GTR+I+G (base frequencies: A 0.24763, C 0.21994, G 0.27379, U 0.25864; rate matrix A-C 1.0507, A-G 2.4454, A-U 1.3720, C-G 0.8041, C-U 4.5502, G-U 1.0000) with the proportion of invariable sites (I = 0.6067) and gamma shape parameter (G = 0.5289) was chosen, which was calculated as the best model by the automated model selection tool implemented in PAUP. The branches in bold are highly supported in all analyses (Bayesian values > 0.95 calculated with PHASE and MrBayes; bootstrap values > 70% calculated with PAUP using maximum likelihood, neighbor-joining, maximum parsimony, and RAxML using maximum likelihood). The sister group of the *Oltmannsiellopsis* clade was chosen as the outgroup. The clade designations were given after the represented genera. The newly sequenced strains were highlighted in bold.

The SSU and ITS rDNA were sequenced after DNA extraction, and PCR protocols were published in Darienko et al. [6]. The new sequence data are available in the EMBL, GenBank, and DDBJ sequence databases under the accession numbers listed in Table 1. SSU rRNA sequences were manually aligned according the secondary structure of SAG 9.90 *Chlorocystis cohnii* (Figure S1). The strains designations and EMBL/GenBank accession numbers of all published sequences used in the phylogenetic analyses are given in Figure 1. Phylogenetic trees were inferred using distance (neighbor-joining), maximum parsimony, and maximum likelihood criteria using PAUP* version 4.0a (build 169; [36]). Two data sets were used: (i) a large SSU alignment of 77 taxa of representatives belonging to the Ulvophyceae *s.str.* with 1775 unambiguously aligned positions, and (ii) a smaller SSU+ITS data set consisting of 33 coccoid and sarcinoid taxa with 2414 unambiguously aligned positions. To decide on the evolutionary model which best fitted to our data, we used the Automated Model Selection tool implemented in PAUP. The settings of the best models are given in the legends of the figures. The robustness of the tree topologies as well as statistical significance were proven by different Bayesian and bootstrap analyses (1000 replicates). The following methods were used for the phylogenetic analyses: distance, maximum parsimony, maximum likelihood, and Bayesian inference. Programs used included PAUP version 4.0a169 [36], RAxML version 8.2.12 [37], MrBayes version 3.2.7a [38], and PHASE package 2.0 [39,40,41,42,43].

### 2.3. Secondary Structure Analyses for Species Delimitation and Distribution Pattern

The secondary structures of ITS-2 sequences were folded using the computer program mfold [44] and analyzed with the ITS-2/CBC approach (based on compensatory base changes in the conserved region of ITS-2), which is described in detail in Darienko & Pröschold [45] for non-marine ulvophytes.

To obtain an overview of the distribution, we used the V4 region of the SSU rDNA and the ITS-2 sequences for searching of entries in GenBank. The V4 and the ITS-2 haplotypes were used for the BLASTn searches (V9: 100% coverage, 100% identity, and ITS-2: 100% coverage, >97% identity; [46]). The metadata of the haplotypes (geographical origin and habitat) are summarized in the Supplementary Materials. To construct the haplotype networks, we used the TCS network tool [47,48] implemented in PopART [49] for three genera *Desmochloris*, *Chlorocystis,* and *Sykidion*.

## 3. Results

### 3.1. Molecular Phylogeny of the Ulvophyceae Based on SSU rDNA Sequences

The initial goal of this study was to present a comprehensive analysis of the phylogeny and taxonomy of coccoid and sarcinoid green algae found in marine habitats. We investigated the available strains of the genera *Chlorocystis* and *Halochlorococcum* (including the authentic strains of *Halochlorococcum dilatatum* Kornmann & Sahling ex Guiry, *H. operculatum* Kornmann & Sahling ex Guiry, and *H. tenue* Kornmann & Sahling ex Guiry), isolated from different marine origins (see Table 1). Both these genera are characterized by a vegetative cell with lobated (*Chlorocystis*) or reticulated chloroplast (*Halochlorococcum*), formation of quadriflagellated zoospores, and “*Codiolum*”-stages during sexual reproduction [16]. To test the significance of these morphological characteristics, we also added strains with sarcinoid organization (*Desmochloris*) and coccoid taxa forming biflagellated zoospores (*Pseudoneochloris*). Phylogenetic analyses of the SSU rDNA sequences have revealed that all investigated strains belong to three lineages of the Ulvophyceae. The sarcinoid strains formed a highly supported sister clade to a clade consisting entirely of taxa previously assigned as *Chlorocystis* and *Halochlorococcum*. Both groups represented the Chlorocystidales consisting of both the genera *Desmochloris* and *Chlorocystis* (Figure 1). The coccoid strains producing biflagellated zoospores represented their own lineage among the Ulvophyceae (Sykidiales ordo nov.; see below). To this clade belonged the authentic strain of *Pseudoneochloris marina* and three taxa previously assigned as unidentified *Chlorococcum* or *Pseudoneochloris*.

Our analyses revealed that the assignment of these coccoids to the so-called *Chlorocystis* group of Ulotrichales by O’Kelly & Floyd [29] is not supported and that they represent, together with the genus *Desmochloris,* their own lineage. The order Ulotrichales *sensu* Mattox & Stewart [9] is also not supported by Bayesian and bootstrap analyses. In contrast, the order Ulvales, also established by Mattox & Stewart [9], received support in all analyses. However, the coccoid and sarcinoid taxa are clearly separated from the filamentous and parenchymatous species. Only the phylogenetic position of the coccoid strain MBIC 10461 (AB058346), designated as coccoid ulvophyte, remained unresolved.

**Figure 2:**
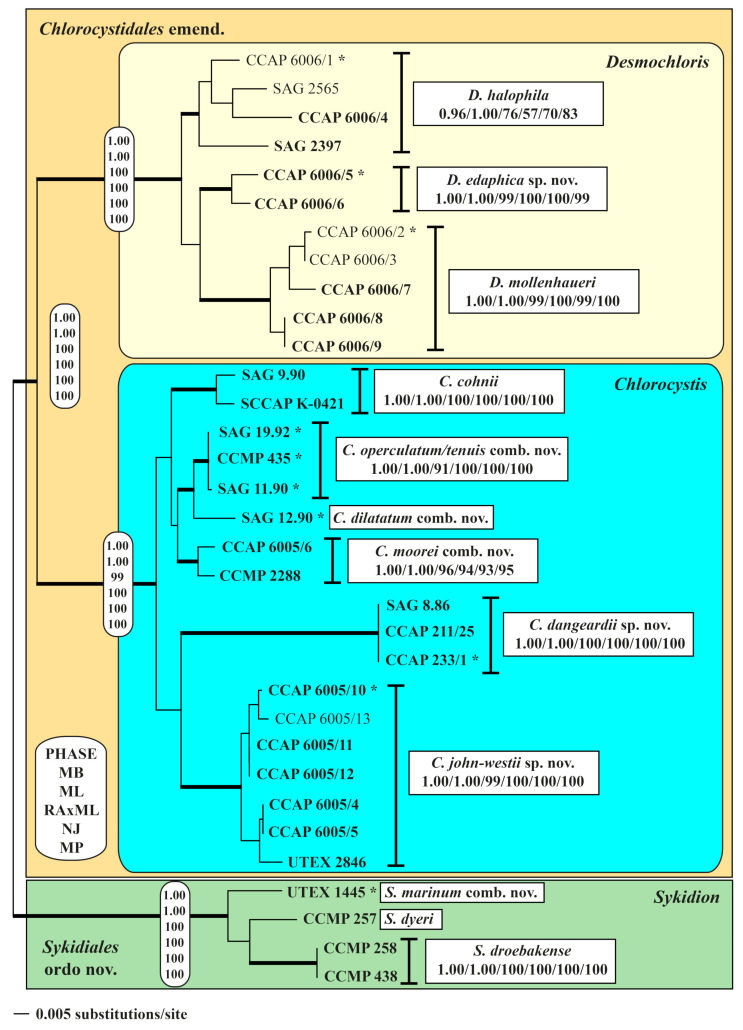
Molecular phylogeny of the Chlorocystidales and Sykidiales based on SSU and ITS rDNA sequence comparisons. The phylogenetic trees shown were inferred using the maximum likelihood method based on the data sets (2410 aligned positions of 33 taxa) using PAUP 4.0a build169. For the analyses, the best model was calculated by the automated model selection tool implemented in PAUP. The setting of the best model was given as follows: SYM+I+G (base frequencies: equal; rate matrix A-C 1.1990, A-G 2.2173, A-U 1.7504, C-G 0.7565, C-U 4.3696, G-U 1.0000) with the proportion of invariable sites (I = 0.7119) and gamma shape parameter (G = 0.4903). The branches in bold are highly supported in all analyses (Bayesian values > 0.95 calculated with PHASE and MrBayes; bootstrap values > 70% calculated with PAUP using maximum likelihood, neighbor-joining, maximum parsimony, and RAxML using maximum likelihood). The authentic strains are highlighted with an asterisk behind the strain number.

To obtain a better resolution among the investigated strains, we analyzed the concatenated data set of SSU and ITS. As demonstrated in Figure 2, the three genera *Desmochloris*, *Chlorocystis,* and *Sykidion* were highly supported in all Bayesian and bootstrap analyses. Within *Desmochloris*, three clades could be revealed. Six and three lineages were discovered in *Chlorocystis* and *Sykidion*, respectively. All subdivisions in these genera are highly supported in all phylogenetic analyses.

### 3.2. ITS-2 Secondary Structures and the Usage of ITS-2/CBC Approach for Species Delineation

**Figure 3:**
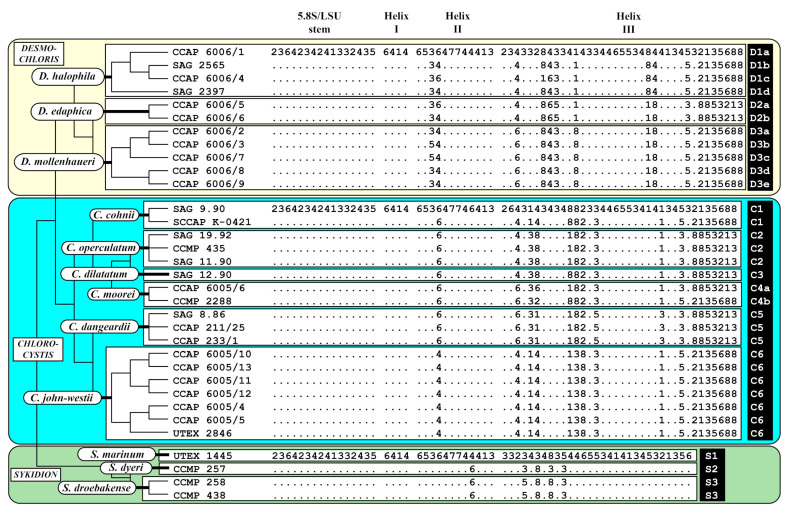
Comparison of the conserved region of ITS-2 among the species of *Desmochloris*, *Chlorocystis,* and *Sykidion*. Extraction of this region and translation into a number code for its usage as barcode. Number code for each base pair: 1 = A–U; 2 = U–A; 3 = G-C; 4 = C–G; 5 = G•U; 6 = U•G; 7 = mismatch; 8 = deletion, single or unpaired bases.

As demonstrated in Figure 2, the genera were subdivided into lineages. This raises the question of if these lineages represent species. Therefore, we analyzed the ITS-2 secondary structures of all investigated strains and used the ITS-2/CBC approach introduced by Darienko & Pröschold [45] for comparison. The secondary structures presented in Figures S2–S4 were highly conserved among the strains. Among the three helices (I-III, IV is missing), only the loops of the helices I and II are variable (highlighted in white boxes). The base pairs of the conserved regions translated into number code showed 17 variable base pair positions in *Desmochloris*, 17 in *Chlorocystis*, and five in *Sykidion* (Figure 3). Each highly supported lineage in Figure 2 could be distinguished by a unique ITS-2 barcode, with few minor changes within species. The three lineages of *Desmochloris* and *Sykidion* are only separated by HCBCs, however, the phylogenetic analyses showed a high support of the separation into three species, respectively. In contrast, the six lineages of *Chlorocystis* are separated by CBCs and HCBCs. As a consequence, all lineages represent species of the *Desmochloris*, *Chlorocystis,* and *Sykidion*.

### 3.3. Distribution of Desmochloris, Chlorocystis and Sykidion Using BLASTn Search Algorithm

**Figure 4:**
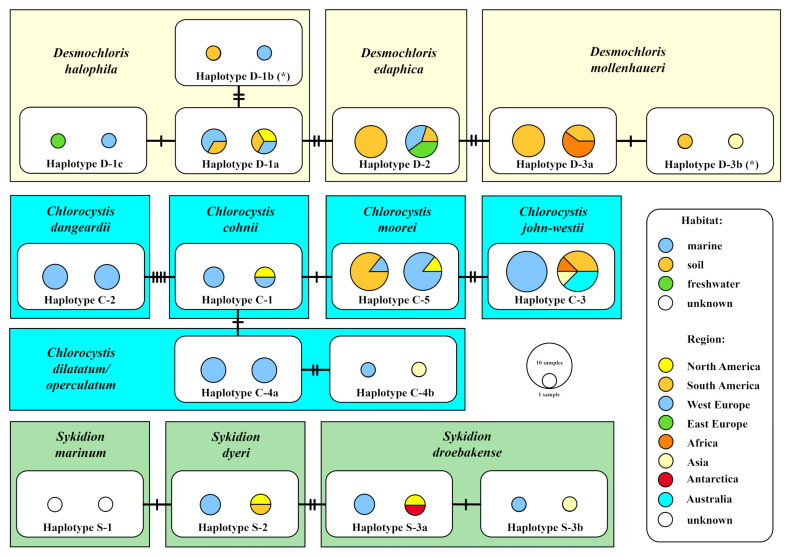
TCS haplotype network inferred from the V4 region of the SSU rDNA sequences of *Desmochloris*, *Chlorocystis,* and *Sykidion*. This network was inferred using the algorithm described by Clement et al. [47,48]. Sequence nodes corresponding to samples collected from different geographical regions and from different habitats. The asterisks mark the haplotypes, which are probably caused by sequencing errors (see Figure 5).

For an overview of the distribution of all species, the V4 and V9 regions of the SSU, commonly used in high-throughput sequence approaches, were proven to be, or not to be, diagnostic for species identification. The V4 region fulfilled this criterion and was used in a BLASTn search in GenBank. In addition, the ITS-2 was also checked, however, no additional entry was found in the BLASTn search. Therefore, we applied the usage of the V4 for finding entries in GenBank. Using this approach (100% coverage, >97% identity), only six additional entries of *Desmochloris*, nine of *Chlorocystis*, and two of *Sykidion* were found (Table S1). The metadata (geographical origin and habitat) belonging to these entries were included with those of the exiting data for our investigated strains to create a dataset for the TCS analyses. As demonstrated in Figure 4, all species could be clearly identified by the V4. Only the authentic strains of *Chlorocystis dilatatum* (SAG 11.90), *C. tenuis* (SAG 19.92), and *C. operculatum* (SAG 11.90) have identical V4 regions. Most species are represented by only one haplotype. For *Desmochloris halophila*, *D. mollenhaueri*, and *Sykidion droebakense*, few haplotypes could be discovered. All species so far known showed no habitat or geographical preference, with two exceptions: (i) the three species of *Desmochloris* occurred in terrestrial habitats, only haplotype D-1a was also found in a marine environment. (ii) The genera *Chlorocystis* and *Sykidion* were exclusively discovered in marine habitats. Interestingly, *Chlorocystis moorei* was also found in saline soils.

**Figure 5:**
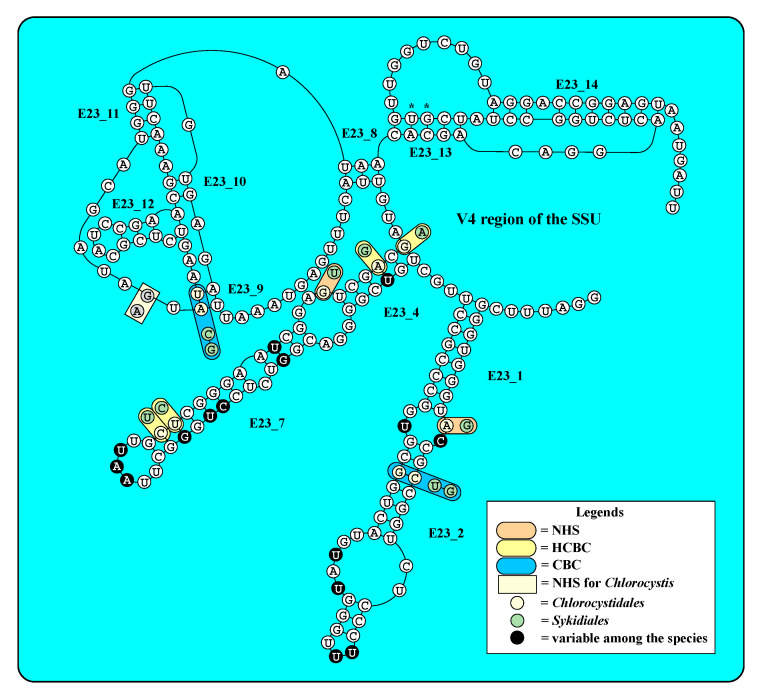
Secondary structure of the hypervariable V4 region (SSU) commonly used for metabarcoding approaches. The asterisks highlight the positions of sequencing mistakes of two entries in GenBank (MH703754 and KF791549; see Table S1).

The haplotypes shown in Figure 4 differed only in few bases in the V4 region of the SSU. To prove where these base differences are located, the secondary structure of V4 was analyzed. The Figure 5 showed the V4 of SAG 9.90 *Chlorocystis cohnii*. The variable base positions appeared at the first part of V4 in the helices E23_1/E23_2, E23_4/E23_7, and E23_9. No other differences occurred in the other helices. Two GenBank entries (MH703754 and KF791549) showed one base difference in E23_13 (marked with an asterisk in Figure 5), respectively, resulting in mismatches in E23_13. These differences presenting haplotypes D-1b and D-3b in Figure 4 are probably sequencing mistakes.

The V4 secondary structure revealed non-homoplasious synaphomorphies (NHSs) and compensatory base changes (HCBCs and CBCs) for the orders Chlorocystidales and Sykidiales, as well as *Chlorocystis*. Two CBCs, four HCBCs, and two NHSs were found to be characteristic for both orders. The genus *Chlorocystis* has one NHS in the spacer between E23_11 and E23_9.

### 3.4. Morphology of the Investigated Strains

**Figure 6:**
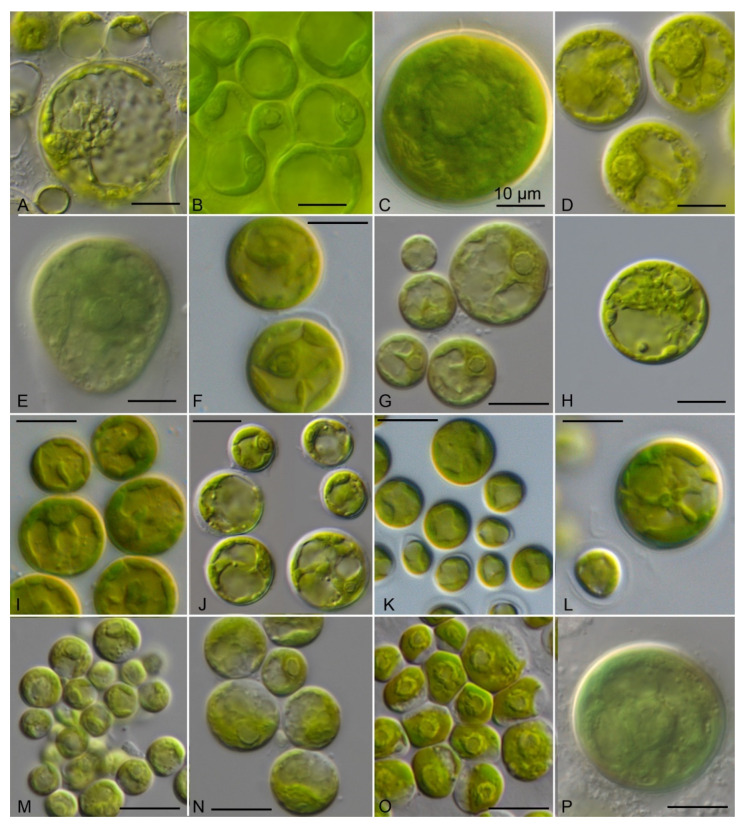
Morphology of the members belonging to the genera *Chlorocystis* and *Sykidion*. (**A**) SAG 9.90 *Chlorocystis cohnii*, (**B**) SAG 19.92 *C. operculatum*, (**C**) SAG 11.90 *C. operculatum*, (**D**) SAG 12.90 *C. dilatatum*, (**E**) CCMP 2288 *C. moorei*, (**F**) CCAP 6005/5 *C. john-westii*, (**G**) CCAP 6005/10 *C. john-westii*, (**H**) CCAP 6005/13 *C. john-westii*, (**I**) CCAP 6005/12 *C. john-westii*, (**J**) SAG 8.86 *C. dangeardii*, (**K**) CCAP 211/25 *C. dangeardii*, (**L**) CCAP 233/1 *C. dangeardii*, (**M**) UTEX 1445 *Sykidion marinum*, (**N**) CCMP 257 *S. dyeri*, (**O**) CCMP 258 *S. droebakense*, (**P**) CCMP 438 *S. droebakense*, scale bar = 10 μm.

The vegetative cells of the coccoid strains showed a spherical to ellipsoid morphology (Figure 6). The chloroplast shape varied from reticulate in *Chlorocystis* (Figure 6A–L) to cup-shaped in *Sykidion* (Figure 6M–P). The morphology of the cells had a high phenotypic plasticity depending on the culture conditions and age of the cultures. The authentic strains SAG 11.90, SAG 12.90, SAG 19.92, and UTEX 1445 were very similar to the original descriptions of the respective species (see [10,16]). Considering the morphology, the strains described as new species (*C. john-westii* sp. nov.; see below) showed similar characteristics as described for the authentic strain of *Halochlorococcum operculatum* (SAG 11.90; [16]), which was already demonstrated by West [50] and West & Braga [51]. The other strains were very difficult to identify at the species level if only the morphology of vegetative cells was known. However, most strains of *Chlorocystis* produced quadriflagellated zoospores. Biflagellated cells were only occasionally observed in *Chlorocystis cohnii* (SAG 9.90) if the tube-dwelling diatom *Berkeleya rutilans* was present, which was described by Kornmann & Sahling [16] for this strain. In contrast, the cultures of *Sykidion* produced only biflagellated zoospores and the strains CCMP 257 and CCMP 258/CCMP 438 could be clearly identified as *S. dyeri* and *S. droebakense*, respectively. The morphology fitted with those of the original descriptions is provided by Wright [11] and Wille [12].

**Figure 7:**
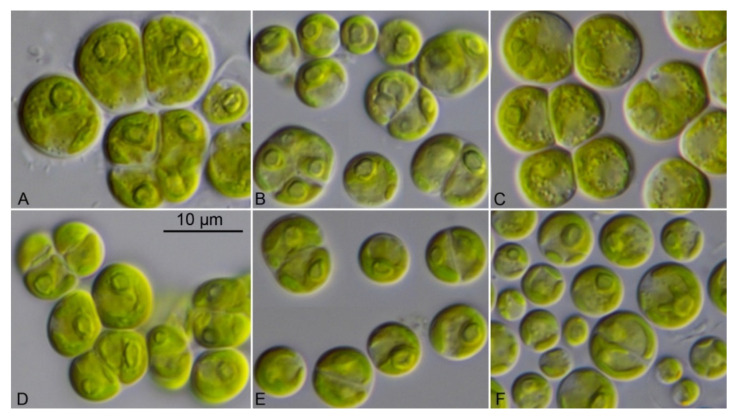
Morphology of the members belonging to the genus *Desmochloris*. (**A**) CCAP 6006/4 *D. halophila*, (**B**) CCAP 6006/5 *D. edaphica*, (**C**) CCAP 6006/6 *D. edaphica*, (**D**) CCAP 6006/7 *D. mollenhaueri*, (**E**) CCAP 6006/8 *D. mollenhaueri*, (**F**) CCAP 6006/9 *D. mollenhaueri*, scale bar for all pictures (**A**–**F**) = 10 μm.

The investigated sarcinoid strains were morphologically identified as *Desmochloris*, a genus which was described in detail in Darienko et al. [15]. As demonstrated in Figure 7, the newly investigated strains CCAP 6006/4 and CCAP 6006/7-9 had similar morphology compared to *D. halophila* (Figure 7A) and *D. mollenhaueri* (Figure 7D–F). The strains CCAP 6006/4 and CCAP 6006/5 (Figure 7B,C) were also very similar in morphology to *D. mollenhaueri*, but differed in sequences as shown above. Therefore, these strains were described as new species, *D. edaphica* sp. nov. (see below).

## 4. Discussion

### 4.1. Two Different Concepts: The Classes Ulvophyceae sensu Mattox and Stewart and Codiolo-phyceae sensu Kornmann and Their Subdivision into Orders

Molecular analyses of the marine coccoid green algae clearly revealed that they are positioned within different lineages of the Ulvophyceae (Figure 1). Traditionally, the Ulvophyceae are recognized by ultrastructural features (counterclockwise basal body orientation [9] and open cell division [52]) and contain the five orders Ulotrichales, Ulvales, Siphonocladales (= Cladophorales), Dasycladales, and Caulerpales (= Bryopsidales) [29,53]. The same ultrastructural features are also present in the class Trebouxiophyceae [54], designated as Pleurastrophyceae in Mattox & Stewart [9] and as Pleurastrales in Sluiman [52]. The definition of the Ulvophyceae and its subdivision into orders have a long history. The concept of this class and its subdivision into the five orders mentioned above was revisited by including the life history in their revised system of the green algae, which led to splitting the Ulvophyceae into five classes: Ulvophyceae *s.str.*, Bryopsidophyceae, Cladophorophyceae, Trentepohliophyceae, and Dasycladophyceae ([55,56] see also details in [2]). Phylogenetic analyses of the SSU rRNA sequences by Watanabe et al. [14] and Zechman et al. [30] have shown that the orders Siphonoclades (= Cladophorales), Dasycladales, and Caulerpales (= Bryopsidales) differ from the orders Ulotrichales and Ulvales, which supported the proposed split of the Ulvophyceae. However, this subdivision into separated classes is not widely accepted and, therefore, all orders are still considered as ulvophytes. Currently, the class Ulvophyceae *s.l.* comprises ten orders (Trentepohliales, Cladophorales, Bryopsidales, Dasycladales, Ulotrichales, Ulvales, Chlorocystidales, Oltmannsiellopsidales, Ignatiales, and Scotinosphaerales) based on phylogenetic analyses of nuclear and plastid gene data [32,45,57,58,59,60,61,62,63,64]. However, the relationship among the ten orders is still not resolved. The reasons for discrepancies within phylogenetic analyses lie in different taxon sampling and the chosen genes. Data sets of many taxa (>more than 100) are only available for single genes, such as SSU or ITS rDNA, and for concatenated data, sets in multiple sequences of the same specimen are only available for a small group of ulvophytes (less than 30). The taxonomic revision at generic and species level remains open and is not resolved.

Another conception for these green algae was proposed by Kornmann [17]. He established a new class, Codiolophyceae, based on the presence of a “*Codiolum*”-stage in the life history; he included the orders Ulotrichales, Monostromatales, Codiolales, and Acrosiphoniales. Kornmann & Sahling [16] added the order Chlorocystidales to the class Codiolophyceae, containing the two coccoid genera, *Chlorocystis* and *Halochlorococcum*, which were included by Floyd & O’Kelly [53] within the Ulotrichales together with *Ulothrix*, *Trichosarcina* (now *Sarcinofilum*; [45]), *Monostroma*, *Acrosiphonia,* and *Urospora*. However, our results showed that the order Chlorocystidales represents its own order and is not a member of the Ulotrichales. Our analyses also revealed that the concept at the class and order level needs to be revised. Kornmann’s Codiolophyceae was not validly described (no Latin diagnosis), but it corresponds with the Ulvophyceae *s.str. sensu* van den Hoek et al. [55,56]. The Ulvophyceae *s.str.* comprises the orders Ulotrichales, Ulvales, Chlorocystidales, Oltmannsiellopsidales, and maybe the orders Ignatiales and Scotinosphaerales, which need further investigation. The five orders of the Codiolophyceae mentioned by Kornmann were only partially supported by our phylogenetic analyses. The Ulotrichales contained taxa, which Kornmann classified as Monostromatales and the members of the Codiolales and Acrosiphoniales belonged to the same order, which formed a separate order (see Figure 1) in contrast to O’Kelly & Floyd [29] and Floyd & O’Kelly [53], who included them in the Ulotrichales. Watanabe et al. [10] added, to the Ulotrichales, *sensu* Floyd & O’Kelly *Pseudoneochloris marina* (UTEX 1445). It was placed between the *Ulothrix* group (consisting *Gloeotilopsis planctonica* and *G. sarcinoidea*, both belong now to the genus *Rhexinema*, *Ulothrix zonata* and *Pseudendoclonium*, now *Chamaetrichon basiliense*) and *Acrosiphonia*. In contrast, in our analyses, the four strains of *Sykidion* (including the authentic strain of *Pseudoneochloris*) form a separate lineage within the Ulvophyceae, which is described below as a new order (see Figure 1).

**Figure 8:**
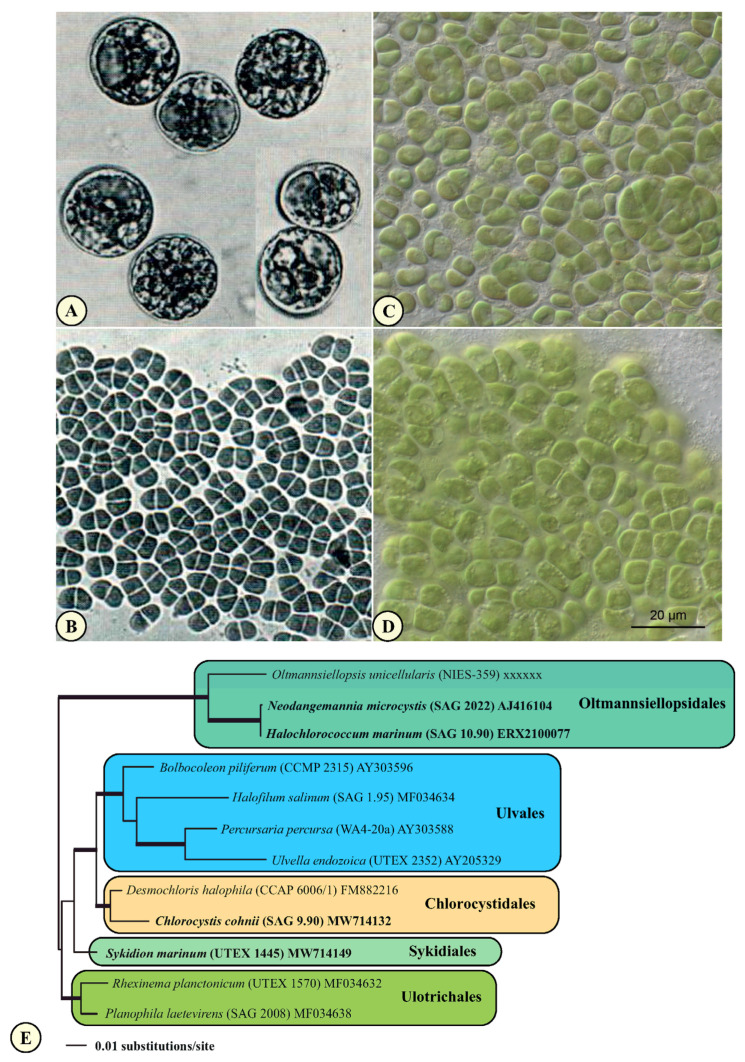
Comparison of the morphology of *Halochlorococcum marinum* (**A**) and *Neodangemannia microcystis* (**B**) published by Kornmann & Sahling [16] with the strains SAG 10.90 (**C**) and SAG 2022 (**D**). (**E**) SSU rDNA phylogeny of the modified dataset used in the transcriptomic study by Gulbrandsen et al. [64]. Scale bar = 20 µm for all pictures (**A**–**D**).

Our sequencing results confirm the proposal of Kornmann & Sahling [16] to exclude *Chlorocystis* and *Halochlorococcum* from the chlorophycean order Chlorococcales, and to transfer them to their newly described order Chlorocystidales (Codiolophyceae), based on studies of their life histories (presence of a “*Codiolum*”-stage). The taxonomic problem arises as follows. The type genus of Chlorocystidales and the type species of *Chlorocystis* present no problem. We could clearly identify *C. cohnii* in our study. The morphology of the strain SAG 9.90 corresponded with the described life cycle demonstrated by Kornmann & Sahling [16]. However, several problems occurred with the second genus, *Halochlorococcum*. No type material of the type species *H. marinum* is available in culture collection. The material of Kornmann & Sahling deposited at SAG under the number SAG 10.90 does not correspond to morphology either presented by the authors nor by Dangeard [65], but fitted in morphology to *Neodangemannia microcystis* (Figure 8A–D). The latter species was originally described as *Ulvella microcystis* by Dangeard [66], the morphology of this species is identical to those demonstrated in Figure 8B,D. Therefore, the transcriptome of SAG 10.90, published in Gulbrandsen et al. [64], represents *Neodangemannia microcystis* and not *Halochlorococcum marinum,* as demonstrated by comparison of the SSU rDNA sequences (see Figure 8E). All other species of *Halochlorococcum* described by Kornmann & Sahling [16] belong to *Chlorocystis* (see Taxonomic consequences below).

In summary, the Ulvophyceae *s.str.* contains the orders Oltmannsiellopsidales, Chlorocystidales, Ulvales, Sykidiales, Acrosiphoniales, and Ulotrichales. The only reason that Mattox and Stewart [9] abandoned Kornmann’s concept of the Codiolophyceae is that the Ulvales are not included because they have no unicellular sporophyte (“*Codiolum*”-stage). However, Bliding [67,68] has shown that *Capsosiphon*, *Ulva,* and *Enteromorpha* (Ulvales) produce a “*Codiolum*”-stage during the zygote germination to the sporophyte. In contrast, for other orders (Trentepohliales, Cladophorales, Bryopsidales, and Dasycladales), no “*Codiolum*”-stage has been reported.

### 4.2. Diversity and Systematics of Marine Coccoid Green Algae

The genus *Chlorocystis* was described by L. Reinhardt in 1885, who found this coccoid green alga in the mucilage tube of diatoms. He investigated the life cycle and morphology of this organism in detail and came to the conclusion that it is a separate genus and is not a member of *Chlorochytrium,* as published by Wright [69]. The main criteria for the separation of *Chlorocystis* were the ecology (marine versus freshwater) and the special morphology of the chloroplast, which is a thin small lobed plate pressed with one large vacuole to the cell wall.

Dangeard [65] described the genus *Halochlorococcum* based on morphological features. The name *Halochlorococcum* was invalid because of the lack of a holotype, and was validated by Guiry [70]. Within this genus, seven species were recognized: *H. marinum* P.J.L. Dangeard ex Guiry, *H. dilatatum* Kornmann & Sahling ex Guiry, *H. moorei* (N.L. Gardner) Kornmann & Sahling ex Guiry, *H. operculatum* Kornmann & Sahling ex Guiry, *H. porphyrae* (Setchell & N.L. Gardner) J.A. West ex Guiry, *H. saccatum* Guillard, H.C.Bold & MacEntee ex Guiry, and *H. tenue* Kornmann & Sahling ex Guiry. The species were discriminated by morphological criteria such as cell size, size and shape of quadriflagellated zoospores, and type of sporangium opening (operculated, non-operculate, with or without rim). The overview of the used morphological criteria is given in West & Braga [51]. The authentic strains SAG 19.92 *H. tenue* and SAG 11.90 *H. operculatum* are identical. Both strains are similar in the size of vegetative cells and quadriflagellated zoospores but differs in sporangia opening, which is wide, circular, operculum with rim in *H. operculatum* and possessing weak operculum in *H. tenue*. The operculum itself seems to be a polyphyletic feature and could be found in at least two lineages. There is no correlation to the host specificity or geographical distribution, which means the same species could be found epiphytic/endophytic and free-living.

### 4.3. Taxonomic Revisions and Diagnoses

As demonstrated above, several taxonomic revisions are required at several ranks. The order Chlorocystidales needs to be emended by adding the genus *Desmochloris* and a new order has to be proposed for the genus *Sykidion*. At the generic level, the genera *Chlorocystis* includes most of the described species originally assigned as *Halochlorococcum* and two newly described species. *Desmochloris edaphica* represents a new species and the genus *Pseudoneochloris* needs to be transferred to the genus *Sykidion*. The formal revisions and descriptions are presented here as follows:

**Chlorocystidales** Kornmann & Sahling, *Helgoländer Meeresunters.* **36**: 24 (descr. prima), 1983.

**Emended Diagnosis**: Chlorophyta unicellular or sarcinoid. Chloroplast parietal or reticulate, with pyrenoid. Asexual reproduction by bi- or quadriflagellated zoospores. Sexual reproduction diplohaplontic, sporophyte as *Codiolum* stage.

**Type family**: Chlorocystidaceae Kornmann & Sahling, *Helgoländer Meeresunters.* **36**: 24 (descr. prima), 1983.

**Emended Diagnosis**: Character as for the order.

**Type genus**: *Chlorocystis* Reinhard 1885 emend.

***Desmochloris*** Watanabe, Kuroda & Maiwa, *Phycologia* **40**: 432, 2001.

**Type species**: *Desmochloris halophila* (Guillard, Bold & MacEntee) Watanabe, Kuroda & Maiwa, *Phycologia* **40**: 432, 2001.

**Synonym**: *Chlorosarcinopsis halophila* Guillard, Bold & MacEntee, *Phycologia* **14**: 17, 1975, fig. 1–8 (descr. et ic. prima, iconotypus).

**Emended Diagnosis**: SSU-ITS sequences (GenBank: FM882216) and ITS-2 Barcode: D1a-d in Figure 3.

**Epitype** (designated here): The authentic strain CCMP 259 cryopreserved in metabolically inactive state at the Bigelow National Center for Marine Algae and Microbiota, East Boothbay, Maine, USA.

***Desmochloris mollenhaueri*** Darienko, Friedl & Pröschold, *Algol. Stud.* **40**: 129, 2009, fig. 3 (descr. et ic. prima, iconotypus).

**Emended Diagnosis**: SSU-ITS sequences (GenBank: FM882217) and ITS-2 Barcode: D3a-e in Figure 3.

**Epitype** (designated here): The authentic strain CCAP 6006/2 cryopreserved in metabolically inactive state at the Culture Collection of Algae and Protozoa, Scottish Association for Marine Science, Oban, UK.

**Figure 9:**
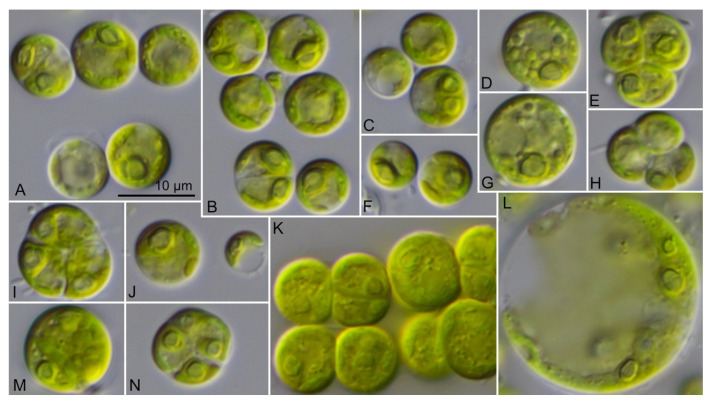
Morphology and phenotypic plasticity of CCAP 6006/5 *Desmochloris edaphica* sp. nov., scale bar = 10 μm for all pictures (**A**–**N**).

***Desmochloris edaphica*** sp. nov. (Figure 9)

**Description**: Vegetative cells are solitary or in packages of 2–8 cells. Single cells are spherical, 6.2 up to 9.9 µm in diameter with a thin cell wall (around 0.5 µm). Chloroplast is cup-shaped with 2–3 incisions covering almost the whole cell. Chloroplast contains one large good visible pyrenoid surrounded by several large starch grains. Young cells contain one large vacuole, which occupies around ¼ of cell volume; in old cells, the vacuole can fill up to half of the cell. Mature vegetative cells are spherical, 12.3 up to 15.9 µm in diameter. Old cells are up to 18.5–26.7 µm in diameter. Cell wall became thicker with age and reached 1.0 µm. Cell packages consist out of 4–8 cells, 9.4 × 9.2–12.6 × 9.9 µm in size. Rarely, tetrads were observed, 8.4–10.2 µm in diameter. Reproduction by zoo- and aplanospores. Sporangia contain usually four spores, which are released by rupturing of the cell wall. Zoospores are biflagellated, with eyespot, cup-shaped chloroplast with a basal pyrenoid. Zoospores after short period of moving become rounded, 5.5–6.5 µm in diameter. Aplanospores after releasing are joined to each other by rests of sporangial cell wall for a long time.

**Diagnosis**: SSU-ITS sequences (GenBank: MW714127) and ITS-2 Barcode: D2a-b in Figure 3.

**Holotype** (designated here): The authentic strain CCAP 6006/5 cryopreserved in metabolically inactive state at the Culture Collection of Algae and Protozoa, Scottish Association for Marine Science, Oban, UK.

**Type locality**: Ukraine, Snake Island, Black Sea, soil sample.

**Etymology**: *edaphica* from Greek “*edaphos*”—soil, ground. 

***Chlorocystis*** Reinhard, *Algological Studies: I. Materials towards the morphology and classification of the algae of Black Sea*, Odessa, p. 14, 1885 *non Chlorocystis* Büttner, *Wiss. Meeresunters. Abt. Kiel N.S*. 12: 123, 1911.

**Synonym**: *Halochloris* Dangeard ex Guiry, *Notulae Algarum* **42**: 2, 2017, *Botaniste* **48**: 68, 1965 (descr. prima).

**Emended Diagnosis**: Vegetative cells solitary or forming aggregates, spherical to subspherical. Chloroplast parietal deeply lobed and become reticulate, with a large central pyrenoid. Reproduction by quadriflagellated zooapores or by biflagellated gametes, which form after fission a unicellular sporophyte (*Codiolum*-stage).

**Type species**: *Chlorocystis cohnii* (Wright) Reinhard.

***Chlorocystis*** *cohnii* (Wright) Reinhard, *Algological Studies: I. Materials towards the morphology and classification of the algae of Black Sea,* Odessa, p. 14, 1885; Wright, *Trans. Roy. Ir. Acad*. 26: 368, 1877, pl. IV, fig. 1–5, pl. V, fig. 1 (descr. et ic. prima, iconotypus) *non Chlorocystis cohnii* (Wright) Reinhard *sensu* Moore (1900).

Basionym: *Chlorochytrium cohnii* Wright, *Trans. Roy. Ir. Acad.* 26: 368, 1877, pl. IV, fig. 1–5, pl. V, fig. 1 (descr. et ic. prima, iconotypus).

Synonym: *Halochloris marinum* Dangeard ex Guiry, *Notulae Algarum* 42: 2, 2017, *Botaniste* 48: 68, 1965, pl. I, fig. 15–25 (descr. et ic. prima, iconotypus).

**Emended Diagnosis**: SSU-ITS sequences (GenBank: MW714132) and ITS-2 Barcode: C1 in Figure 3. 

**Epitype** (designated here): The strain SAG 9.90 permanently preserved in a metabolically inactive state (cryopreservation in liquid nitrogen) in the Culture Collection of Algae, University of Göttingen, Germany.

Comment: This species has been reported from Helgoland, Germany [16,71], Bohuslän, Sweden [72], British Isles [73] and Black Sea, Ukraine [74].

***Chlorocystis dilatatum*** (Kornmann & Sahling ex Guiry) comb. nov. 

**Basionym**: *Halochlorococcum dilatatum* Kornmann & Sahling ex Guiry, *Notulae Algarum* **42**: 1, 2017, *Helgoländer Meeresunters.* **36**: 42, 1983, fig. 23 (descr. et ic. prima, iconotypus).

**Emended Diagnosis**: SSU-ITS sequences (GenBank: MW714139) and ITS-2 Barcode: C3 in Figure 6. 

**Epitype** (designated here): The authentic strain SAG 12.90 permanently preserved in a metabolically inactive state (cryopreservation in liquid nitrogen) in the Culture Collection of Algae, University of Göttingen, Germany.

***Chlorocystis moorei*** (Gardner) comb. nov. 

**Basionym**: *Chlorochytrium moorei* Gardner, *Univ. Calif. Publ. Bot.* **6**: 382, 1917, lectotype BM (Collins, Holden & Setchell, Phyc. Bor.-Amer. No. 565, as *Chlorocystis cohnii* (Wright) Reinh.), *Halochlorococcum* moorei (Gardner) Kornmann & Sahling ex Guiry, *Notulae Algarum* **42**: 1, 2017, *Helgoländer Meeresunters.* **36**: 42, 1983.

**Synonym**: *Chlorochytrium willei* Printz, Die Algenvegetation des Trondhjemsfjordes, *Skr. Norsk. Vid.-Akad. Oslo, 1. Mat.-Nat. Kl.* **5**: 219, 1926, pl. VII, fig. 73–101 (descr. et ic. prima, iconotypus), *Chlorocystis cohnii sensu* Moore, *Bot. Gaz.* **30**: 100–110, 1900, pl. X, fig 1–14, *Chlorochytrium porphyrae* Setchell & Gardner in Gardner, *Univ. Calif. Publ. Bot.* **6**: 379, 1917, holotype: UC 205703, *Halochlorococcum porphyrae* (Setchell & Gardner) West ex Guiry, *Notulae Algarum* **42**: 1–2, 2017, West in West, Smith & McBride, *Botanica Marina* **31**: 304, 1988.

**Emended Diagnosis**: SSU-ITS sequences (GenBank: MW714138) and ITS-2 Barcode: C4a-b in Figure 3. 

**Epitype** (designated here): The authentic strain CCMP 2288 cryopreserved in metabolically inactive state at the Bigelow National Center for Marine Algae and Microbiota, East Boothbay, Maine, USA.

Comment: This species has been reported from the Northern Hemisphere: Helgoland, Germany [16], Trondheim fjord, Norway [75], MA, USA [76], CA, USA [77,78], and British Isles [73]. This species was originally described as different species of *Chlorochytrium*. *C. willei* and *C. porphyrae* differ only slightly in morphology to *C. moorei* (cell wall thickening in *C. willei* and presence of biflagellated isogametes in *C. porphyrae*) and were found as epiphytes of *Blidingia minima* and species of *Porphyra*, respectively. The partial SSU rDNA sequences of *Halochlorococcum porphyrae* (DQ821518, DQ821519 and DQ821520; [79]) were identical to both strains (CCAP 6005/6 and CCMP 2288).

***Chlorocystis operculatum*** (Kornmann & Sahling ex Guiry) comb. nov.

**Basionym**: *Halochlorococcum operculatum* Kornmann & Sahling ex Guiry, *Notulae Algarum* 42: 1, 2017, *Helgoländer Meeresunters.* 36: 40, 1983, fig. 22 (descr. et ic. prima, iconotypus).

Synonym: *Halochlorococcum tenue* Kornmann & Sahling ex Guiry, *Notulae Algarum* 42: 2, 2017, *Helgoländer Meeresunters.* 36: 39, 1983, fig. 21 (descr. et ic. prima, iconotypus).

Emended Diagnosis: SSU-ITS sequences (GenBank: MW714136) and ITS-2 Barcode: C2 in Figure 3. 

**Epitype** (designated here): The authentic strain SAG 11.90 permanently preserved in a metabolically inactive state (cryopreservation in liquid nitrogen) in the Culture Collection of Algae, University of Göttingen, Germany.

Comment: This species originally described as *Halochlorococcum operculatum* corresponds in morphology with *H. tenue* (only differences in cell size and sporangia opening) and is only known from Helgoland, Germany [16]. The other records from Southern Hemisphere also correspond with the type description [50,51], but differ in SSU and ITS rDNA sequences and therefore represent a new species (see below).

**Figure 10:**
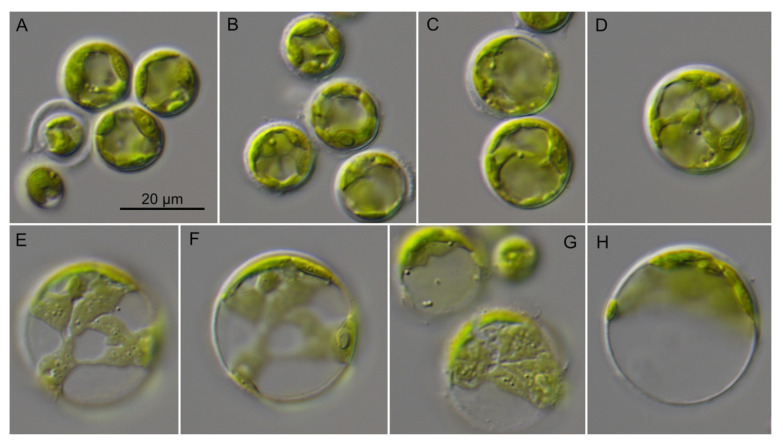
Morphology and phenotypic plasticity of SAG 8.86 *Chlorocystis dangeardii* sp. nov., scale bar = 20 μm for all pictures (**A**–**H**).

***Chlorocystis dangeardii*** sp. nov. (Figure 10)

**Description**: Young vegetative cells are solitary or forming aggregates, spherical or subspherical. Chloroplast parietal deeply lobed and becomes net-like, with a large central pyrenoid and thin cell wall. Young cells 5.2 up to 8.6 µm in diameter. Mature vegetative cells are usually solitary, sometimes could form cell aggregates. Solitary cells are spherical, or subspherical, 12.0–24.0 µm, rarely up to 30.0–42.0 µm in diameter. Chloroplast is reticulate or parietal, deeply lobed, and perforated. Cells contain several large vacuoles, which push the chloroplast to the cell wall. Pyrenoid is central, large, conspicuous, surrounded by several large starch grains. Cell wall thin, around 0.6–0.9 µm and became thicker with age (up to 1.0–1.2 µm). Old cells are spherical with layered and partially granulated cell wall up to 2.0 µm. Sexual reproduction was not observed. Asexual reproduction by producing quadriflagellated zoospores. Zoosporangia 14.6–24.0 µm in diameter and contain 8–32 zoospores. Releasing of zoospores through an apical circular pore. Zoospores are 6.0–7.0 µm long × 3.5–4.0 µm wide, with parietal chloroplast, with pyrenoid and anterior-lateral stigma. The zoospores are released in a mucilage vesicle, which dissolves in several minutes. Zoospores after 10–20 min active movement settle down and become spherical 5.5–6.5 µm in diameter. Young cells retain the stigma for some period of time. 

**Diagnosis**: SSU-ITS sequences (GenBank: MW714142) and ITS-2 Barcode: C5 in Figure 3.

**Holotype** (designated here): The authentic strain CCAP 233/1 cryopreserved in metabolically inactive state at the Culture Collection of Algae and Protozoa, Scottish Association for Marine Science, Oban, UK.

**Type locality**: France, Soulac, from *Ulva* culture, isolated by Izard (1965).

**Etymology**: The species is named in honor of Pierre J. L. Dangeard for his contribution in marine phycology.

**Figure 11:**
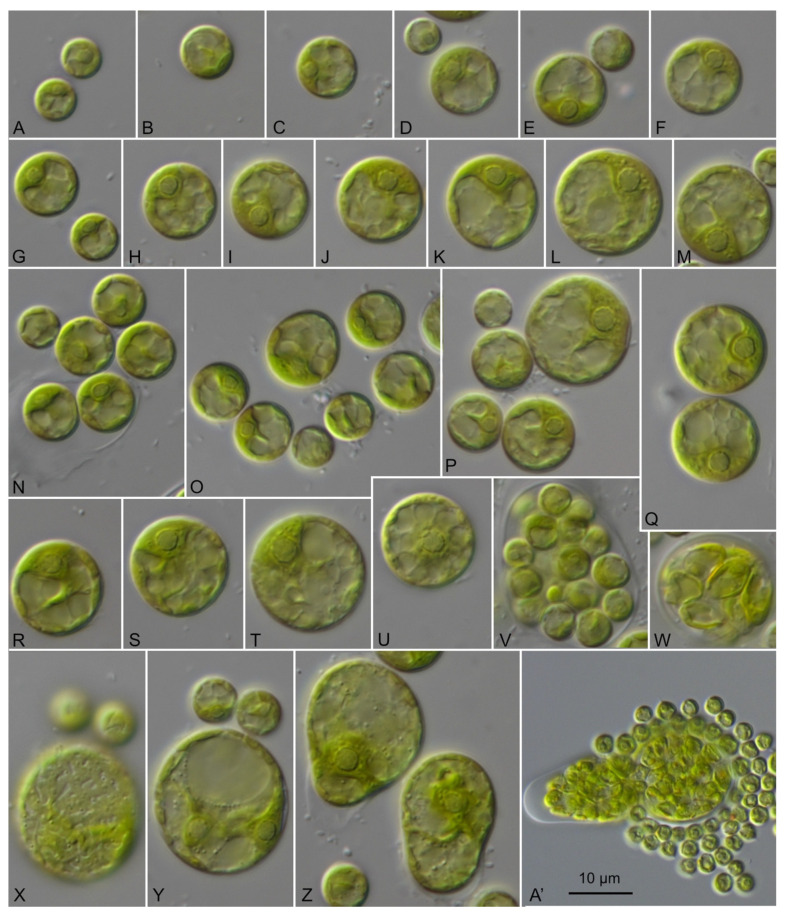
Morphology and phenotypic plasticity of CCAP 6005/10 *Chlorocystis john-westii* sp. nov., scale bar = 10 μm for all pictures (**A**–**A**’).

***Chlorocystis john-westii*** sp. nov. (Figure 11)

**Description**. Young vegetative cells are unicellular or forming clumps, with spherical, broadly ellipsoidal, or sometimes pyriform cell shape. Chloroplast parietal lobed, quickly became reticulate, with large central pyrenoid. Cell wall very thin. Cells contain one large good visible nucleus. Mature vegetative cells are solitary or gathered into crust-like aggregates. Solitary cells are spherical, subspherical, pyriform, or irregular, 12.0–25.0 µm in diameter. Cells in the aggregates are polygonal. Chloroplast is parietal, perforated, or reticulate. Cells contain several large vacuoles, which perforate the chloroplast and sometimes push it to the cell wall. Pyrenoid is central, conspicuous, surrounded by several large starch grains. Cell wall thin, around 0.5 µm and became thicker with age (up to 1.2–2.0 µm). Old cells are spherical or often irregular-saccate with layered cell wall up to 3.0 µm. Sexual reproduction by producing biflagellated gametes. Asexual reproduction by producing quadriflagellated zoospores. Zoosporangia 30–45 µm in diameter and contain 32 or more zoospores. Releasing of zoospores through an apical circular pore with an operculum that remains sometimes connected at one edge, but often breaks down. Zoospores are 6.0–7.0 µm long × 3.5–4.0 µm wide, with parietal chloroplast, with pyrenoid and anterior-lateral eyespot. The zoospores are released in mucilage vesicle, which dissolves in several minutes. Zoospores are positive phototactic and after 10–20 min active movement settle down and become spherical 5.5–6.5 µm in diameter. Young cells retain the eyespot for some period of time. 

**Diagnosis**: SSU-ITS sequences (GenBank: MW714143) and ITS-2 Barcode: C6 in Figure 3.

**Holotype** (designated here): The authentic strain CCAP 6005/10 cryopreserved in metabolically inactive state at the Culture Collection of Algae and Protozoa, Scottish Association for Marine Science, Oban, UK.

**Type locality**: Peru, Tumber Province, Pacific Ocean, epiphyte on *Bostrychia radians*.

**Etymology**: The species is named in honor of John A. West for his contribution in marine phycology.

Comment: The alga occurs as an epiphyte/endophyte on different seaweeds such as *Bostrychia*, *Blidingia*, and *Porphyra* and probably has a cosmopolitan distribution in the southern hemisphere. No records in the northern hemisphere are known, however, this needs further studies.

**Sykidiales** ordo nov.

**Description**: Chlorophyta unicellular. Chloroplast cup-shaped, parietal or reticulate, with pyrenoid. Asexual reproduction by biflagellated zoospores.

**Type family**: Sykidiacaeae fam. nov.

**Description**: Character as for the order.

**Type genus**: *Sykidion* Wright 1881 emend.

***Sykidion*** Wright, *Trans. Roy. Ir. Acad.* **28**: 29, 1881 emend.

**Basionym**: *Pseudoneochloris* Watanabe, Himizu, Lewis, Floyd & Fuerst, *J. Phycol.* **36**: 603, 2000 (descr. prima).

**Emended Diagnosis**: Vegetative cells are solitary, spherical, slightly flattened, or polygonal. Cells are uninucleate with cup-shaped or saucer-shaped chloroplast containing a pyrenoid surrounded by several large starch grains. Cell wall is thin in young cells and became slightly thicker (around 1 µm) in old cells. Vegetative cells possess one, up to several, large vacuoles occupying around half of cell volume. Reproduction by aplano- or zoospores. Releasing of zoospores by apical, one side rupturing of sporangia. Zoospores are released in mucilage envelope, which disappears in several minutes after liberation. Zoospores are without cell wall or scales. Zoospores are biflagellated with counterclockwise basal bodies orientation. Sexual reproduction was not observed, but *Codiolum*-stages could be observed in old cultures.

**Type species**: *Sykidion dyeri* Wright 1881 emend.

***Sykidion dyeri*** Wright, *Trans. Roy. Ir. Acad.* **28**: 29, 1881, pl. II, fig. 5, (descr. et ic. prima, iconotypus), Type: TCD. Howth near Dublin, Ireland.

**Figure 12:**
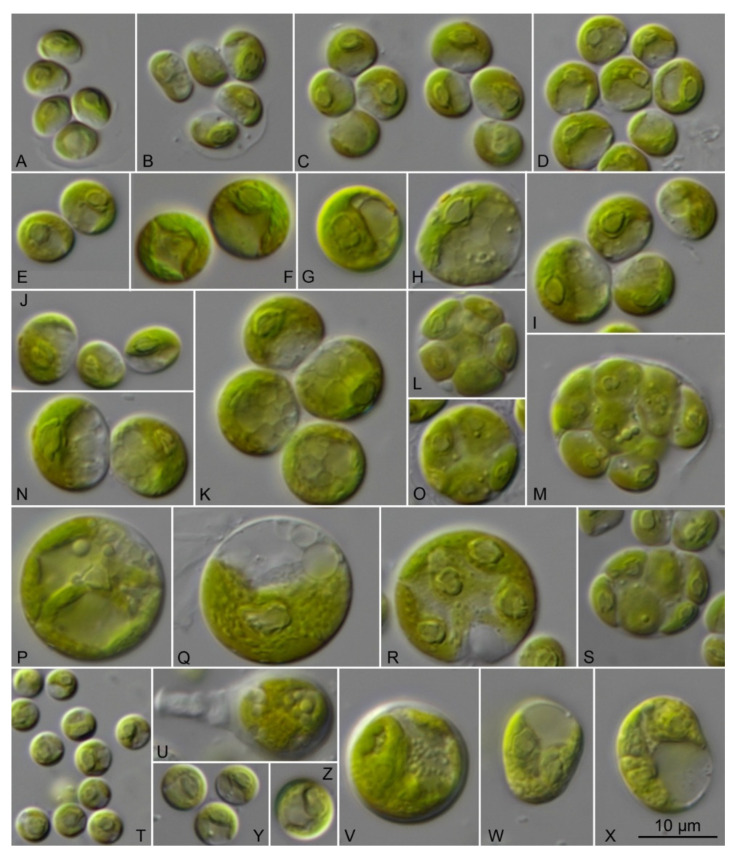
Morphology and phenotypic plasticity of CCMP 257 *Sykidion dyeri*, scale bar = 10 μm for all pictures (**A**–**Z**).

**Emended description** (Figure 12): Young cells are 6.7 × 5.8 µm up to 12.5 × 12.1 µm round-flattened, solitary or forming a thin biofilm. The cells are uninucleate, with cup-shaped chloroplast containing very good visible pyrenoid surrounded by several starch grains. Cells have one large vacuole and a thin smooth cell wall. Mature vegetative cells are roundish, irregular oval, saccate, reniform. Cell size fluctuated among 16.1 × 16.6 µm up to 31.2 × 23.8 µm. Cell wall of the mature cells sometimes is layered. The inner layer is thin (around 0.7 µm). Outer layer is often partially sticking on cells and is up to 2 µm. Cells contain several large vacuoles and parietal chloroplast with pyrenoid surrounded by several starch grains. Reproduction by zoospores or aplanospores. Zoospores are without cell wall, parietal chloroplast with pyrenoid and eyespot. After moving the cells become round 6.5–7.0 µm in diameter. Zoosporangia contain 4–8 spores, 20.6 × 14.8 or 13.8–14.8 µm if spherical. Aplanosporangia contain 4–8 cells and after releasing often stay together in form of tetrads. The zoospores and aplanospores are releasing by rupture at one side of cell wall. SSU-ITS sequences (GenBank: MW714150) and ITS-2 Barcode: S2 in Figure 3.

**Epitype** (designated here): The strain CCMP 257 cryopreserved in metabolically inactive state at the Bigelow National Center for Marine Algae and Microbiota, East Boothbay, Maine, USA.

***Sykidion droebakense*** Wille, *Vidensk. Skr., Math.-Naturvid. Kl.* 1900, 6: 7, 1901, Table I, fig. 1–16, (descr. et ic. prima, iconotypus).

**Figure 13:**
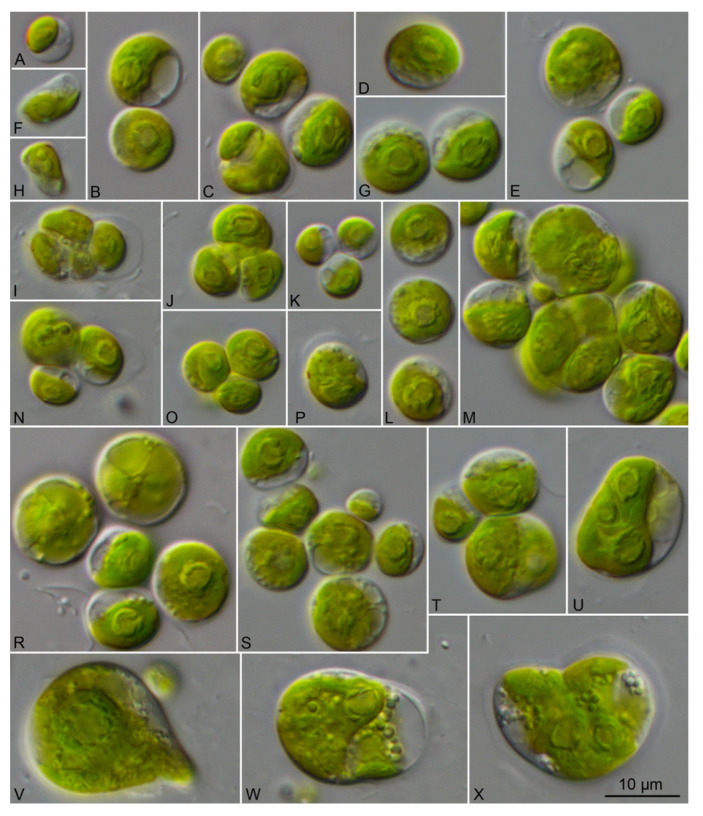
Morphology and phenotypic plasticity of CCMP 258 *Sykidion droebakense*, scale bar = 10 μm for all pictures (**A**–**P**,**R**–**X**).

**Emended description** (Figure 13): Vegetative cells are free floating or attached to the substrate or epiphytic on marine seaweeds (like *Rhizoclonium* or *Cladophora* species) and forming thin biofilms. Cells are spherical in case of free-floating or slightly flattened or polygonal-irregular if they form biofilms. Young vegetative cells 5.5–9.0 µm in diameter or 5.5 × 5.0 up 7.8 × 6.6 µm if flattened. They have thin cell walls (0.5 µm) and cup-shaped chloroplasts with single pyrenoids. Cells are solitary or in groups of 4 or 8 cells. Mature cells are 8.5–18.7 µm in diameter or 9.7 × 9.5 up 12.9 × 13.9 if flattened. Chloroplast is thick and covering a large part of the cell, containing many starch grains. Pyrenoid is large, good visible, oval to spherical, surrounded by several starch grains. Cell nucleus is clearly visible and located in the upper part of the cell. Old cells sometimes have one side of the cell wall a thickening on one side of the cell wall (similar to a plug, which is oriented inside, up to 4.2 × 5.0 µm). Reproduction by zoospores and aplanospores production. Sporangia 11.6 × 15.0 µm or 10.0–13.0 µm in diameter. Small cell wall plugs (1.9 × 1.4 µm) are observed in some sporangia. Zoospores are without cell wall, biflagellated with eyespot. After short period of moving the zoospores become round. Zoospores and aplanospores produced in numbers of 4–16 cells. Releasing of spores at one side by rupturing of the sporangium cell wall. Spores released in mucilage envelope. The remains of the sporangial wall stay for long time in culture and slowly dissolve. Partially dissolving remains of the sporangial cell wall kept the young vegetative cells together in groups. SSU-ITS sequences (GenBank: MW714151) and ITS-2 Barcode: S3 in Figure 3.

**Epitype** (designated here): The strain CCMP 258 cryopreserved in metabolically inactive state at the Bigelow National Center for Marine Algae and Microbiota, East Boothbay, Maine, USA.

***Sykidion marinum*** (Watanabe, Himizu, Lewis, Floyd & Fuerst) comb. nov.

**Basionym**: *Pseudoneochloris marina* Watanabe, Himizu, Lewis, Floyd & Fuerst, *J. Phycol.* 36: 603, 2000, figs 1–25 (descr. et ic. prima, iconotypus).

**Emended diagnosis**: SSU-ITS sequences (GenBank: MW714149) and ITS-2 Barcode: S1 in Figure 3.

**Epitype** (designated here): The authentic strain UTEX 1445 cryopreserved in metabolically inactive state at the Culture Collection of Algae at the University of Texas, Austin, Texas, USA.

## Figures and Tables

**Table 1:** Origin of strains including the revised species names and accession numbers investigated in this study.

Strain	Species	Origin	Accession ^1^
***Chlorocystis***			
SAG 9.90	*C. cohnii*	Germany, Helgoland, from tube jelly of colony-forming *Berkelya rutilans*	**MW714132**
SCCAP K-0421	*C. cohnii*	Greenland, Godthåbsfjorden, Kapisillit, endophyte on *Polysiphonia violacea*	**MW714133**
SAG 8.86	*C. dangeardii*	UK, North Wales, oyster breeding tank at Conway	**MW714140**
CCAP 233/1 ^2^	*C. dangeardii*	France, Ulva culture from Soulac	**MW714142**
CCAP 211/25	*C. dangeardii*	UK, England, Cornwall, Henn Point, Tamar Estuary	**MW714141**
SAG 12.90	*C. dilatatum*	Germany, Helgoland, littoral rock pool	**MW714139**
CCAP 6005/4	*C. john-westii*	Brazil, São Paulo, Ilha do Cardoso, Rio Pereque, endophyte in *Bostrychia calliptera*	**MW714146**
CCAP 6005/5	*C. john-westii*	Australia, Queensland, Gladstone, endophyte in *Bostrychia moritziana*	**MW714147**
CCAP 6005/10	*C. john-westii*	Peru, Tumbes, Puerto Pizzaro, endophyte in *Bostrychia radicans*	**MW714143**
CCAP 6005/11	*C. john-westii*	Australia, Queensland, Bowling Green Bay, endophyte in *Bostrychia bispora*	**MW714144**
CCAP 6005/12	*C. john-westii*	Australia, Queensland, Bowling Green Bay, endophyte in *Bostrychia moritziana*	**MW714145**
CCAP 6005/13	*C. john-westii*	Madagascar, Chenal d’Ampanarata, Belo sur Mer, epiphyte on *Bostrychia pinnata*	MK541803
UTEX 2846	*C. john-westii*	Brazil, Maranhão, Parra Açu, endophyte in *Bostrychia montagnei*	**MW714148**
CCAP 6005/6	*C. moorei*	Germany, Helgoland, epiphyte on *Blidingia minima*	**MW714137**
CCMP 2288	*C. moorei*	USA, Washingtion, Friday Harbor, San Juan Isand	**MW714138**
SAG 11.90	*C. operculatum*	Germany, Helgoland from oyster-shell in a littoral pool	**MW714136**
SAG 19.92= CCMP 435 ^2^	*C. operculatum*	France, Ulva culture from Soulac	**MW714134** **MW714135**
***Desmochloris***			
CCAP 6006/5	*D. edaphica*	Ukraine, Snake Island, Black Sea, soil	**MW714127**
CCAP 6006/6	*D. edaphica*	Chile, Atacama, biological soil crust	**MW714128**
CCAP 6006/1	*D. halophila*	USA, MA, Martha’s Vineyard, Great Pond	FM882216
SAG 2565	*D. halophila*	Germany, Island Rügen, the coast of the Baltic Sea, sand dunes	MH703754
SAG 2397	*D. halophila*	Germany, Franconian Alb, Deinschwanger Bach, biofilm on rock surface	**MW714126**
CCAP 6006/4	*D. halophila*	Chile, Atacama, soil	**MW714125**
CCAP 6006/2	*D. mollenhaueri*	South Africa, Flaminkvlakte, Van Rhynsdorp, biological soil crust	FM882217
CCAP 6006/3	*D. mollenhaueri*	South Africa, Groot Derm-Yellow Dune 10, biological soil crust	FM882218
CCAP 6006/7	*D. mollenhaueri*	South Africa, Koeroegap Vlakte, biological soil crust	**MW714129**
CCAP 6006/8	*D. mollenhaueri*	Chile, Atacama, biological soil crust	**MW714130**
CCAP 6006/9	*D. mollenhaueri*	Chile, Atacama, biological soil crust	**MW714131**
***Sykidion***			
CCMP 258	*S. droebakense*	Canada, BC, Vancouver Island, from South Long Beach	**MW714151**
CCMP 438 ^3^	*S. droebakense*	Antarctica, from Palmer Station	**MW714152**
CCMP 257	*S. dyeri*	USA, CT, Milford, from tank	**MW714150**
UTEX 1445	*S. marina*	origin unknown	**MW714149**

^1^ New SSU and ITS rDNA sequences of this study written in bold. ^2^ Strains isolated from the same enrichment culture. ^3^ This strain originated from CCMP contains two different ulvophycean species. We investigated only the coccoid form. The filamentous species belong to another group (data not shown).

## Data Availability

The sequence data are available under the given accession numbers in GenBank.

## Supplementary Materials

The following are available online at https://www.mdpi.com/article/10.3390/microorganisms9081586/s1, Figure S1: SSU rRNA secondary structure of SAG 9.90 *Chlorocystis cohnii*, Figure S2: ITS-2 rRNA secondary structures of the strains belonging to the genus *Desmochloris*. The line structures were created using the program PseudoViewer3 (http://pseudoviewer.inha.ac.kr). The variable regions are highlighted in white boxes, Figure S3: ITS-2 rRNA secondary structures of the strains belonging to the genus *Chlorocystis*. The line structures were created using the program PseudoViewer3 (http://pseudoviewer.inha.ac.kr). The variable regions are highlighted in white boxes, Figure S4: ITS-2 rRNA secondary structures of the strains belonging to the genus *Sykidion*. The line structures were created using the program PseudoViewer3 (http://pseudoviewer.inha.ac.kr). The variable regions are highlighted in white boxes, Table S1: GenBank entries (accession numbers) found by BLAST N search (100% coverage; 100% identity) using the V4 region of the SSU rDNA of each species. To each entry the geographical origin and after the habitat are given. The investigated strains are marked in green.
