# Supplementary material for: Molecular Phylogeny of Unicellular Marine Coccoid Green Algae Revealed New Insights into the Systematics of the Ulvophyceae (Chlorophyta)
Source: Microorganisms. 2021 Jul 26;9(8):1586. doi: 10.3390/microorganisms9081586 (PMC8401757; doi:10.3390/microorganisms9081586)
Supplement: Supplementary file 1 [file microorganisms-09-01586-s001.zip › Figure_S2.pdf]

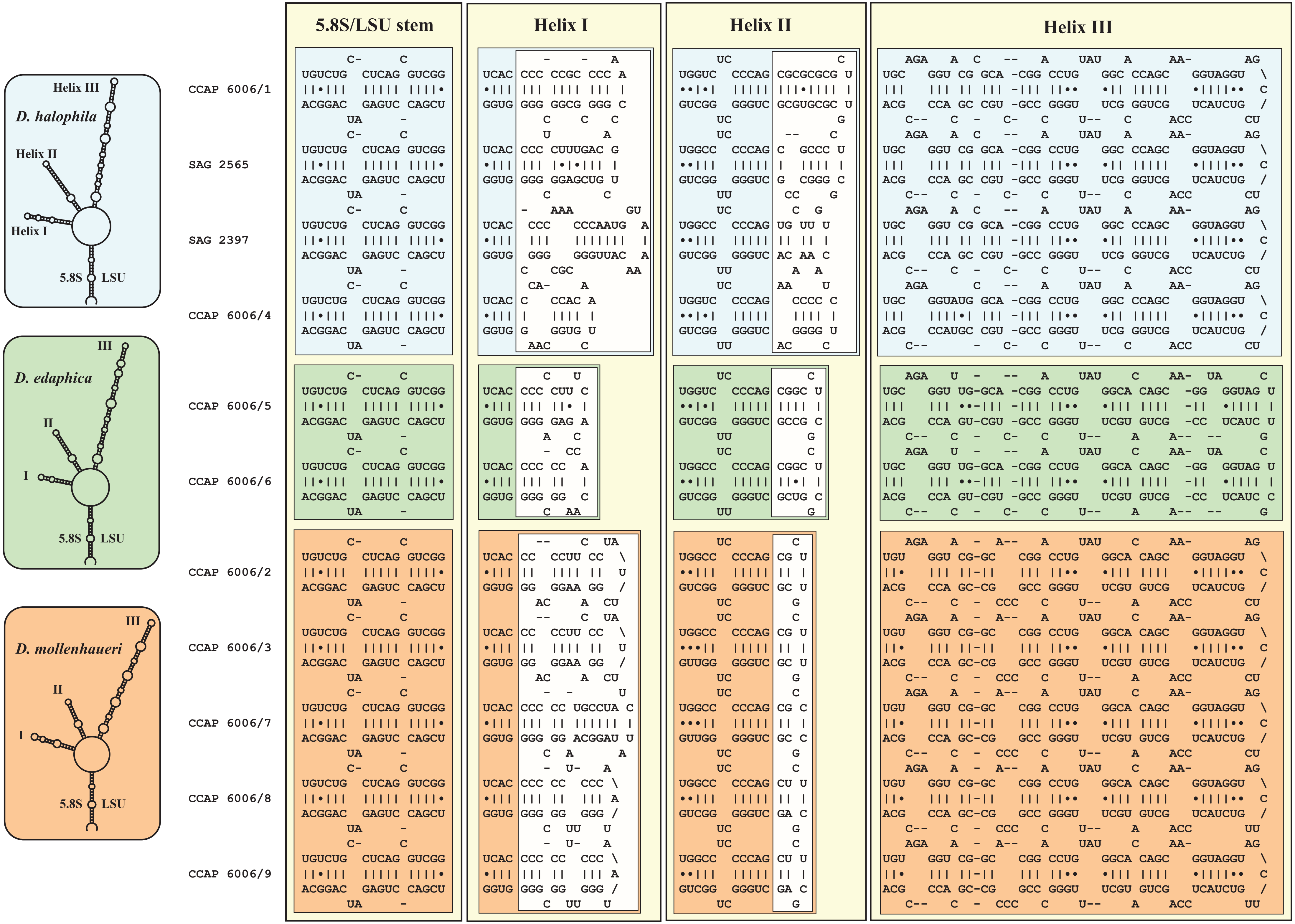
5.8S/LSU stem
Helix I
Helix II
Helix III
D. halophila
Helix III
Helix II
Helix I
5.8S
LSU
D. edaphica
III
II
I
5.8S
LSU
D. mollenhaueri
III
II
I
5.8S
LSU
CCAP 6006/1
SAG 2565
SAG 2397
CCAP 6006/4
CCAP 6006/5
CCAP 6006/6
CCAP 6006/2
CCAP 6006/3
CCAP 6006/7
CCAP 6006/8
CCAP 6006/9
