# Supplementary material for: Molecular Phylogeny of Unicellular Marine Coccoid Green Algae Revealed New Insights into the Systematics of the Ulvophyceae (Chlorophyta)
Source: Microorganisms. 2021 Jul 26;9(8):1586. doi: 10.3390/microorganisms9081586 (PMC8401757; doi:10.3390/microorganisms9081586)
Supplement: Supplementary file 1 [file microorganisms-09-01586-s001.zip › Figure_S3.pdf]

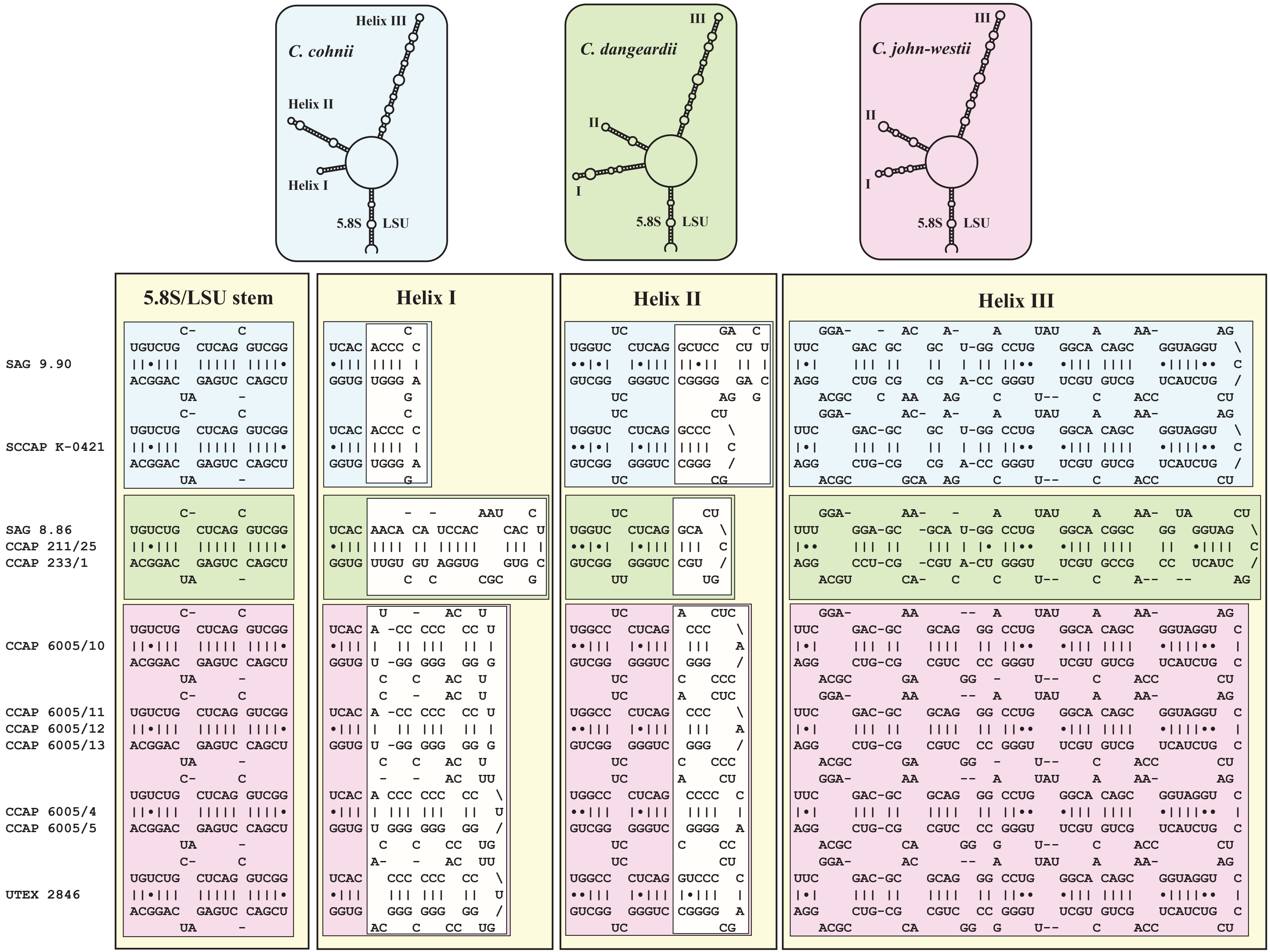

C. cohnii
Helix I
Helix II
Helix III
5.8S
LSU
C. dangeardii
I
II
III
5.8S
LSU
C. john-westii
I
II
III
5.8S
LSU
5.8S/LSU stem
Helix I
Helix II
Helix III
SAG 9.90
SCCAP K-0421
SAG 8.86
CCAP 211/25
CCAP 233/1
CCAP 6005/10
CCAP 6005/11
CCAP 6005/12
CCAP 6005/13
CCAP 6005/4
CCAP 6005/5
UTEX 2846

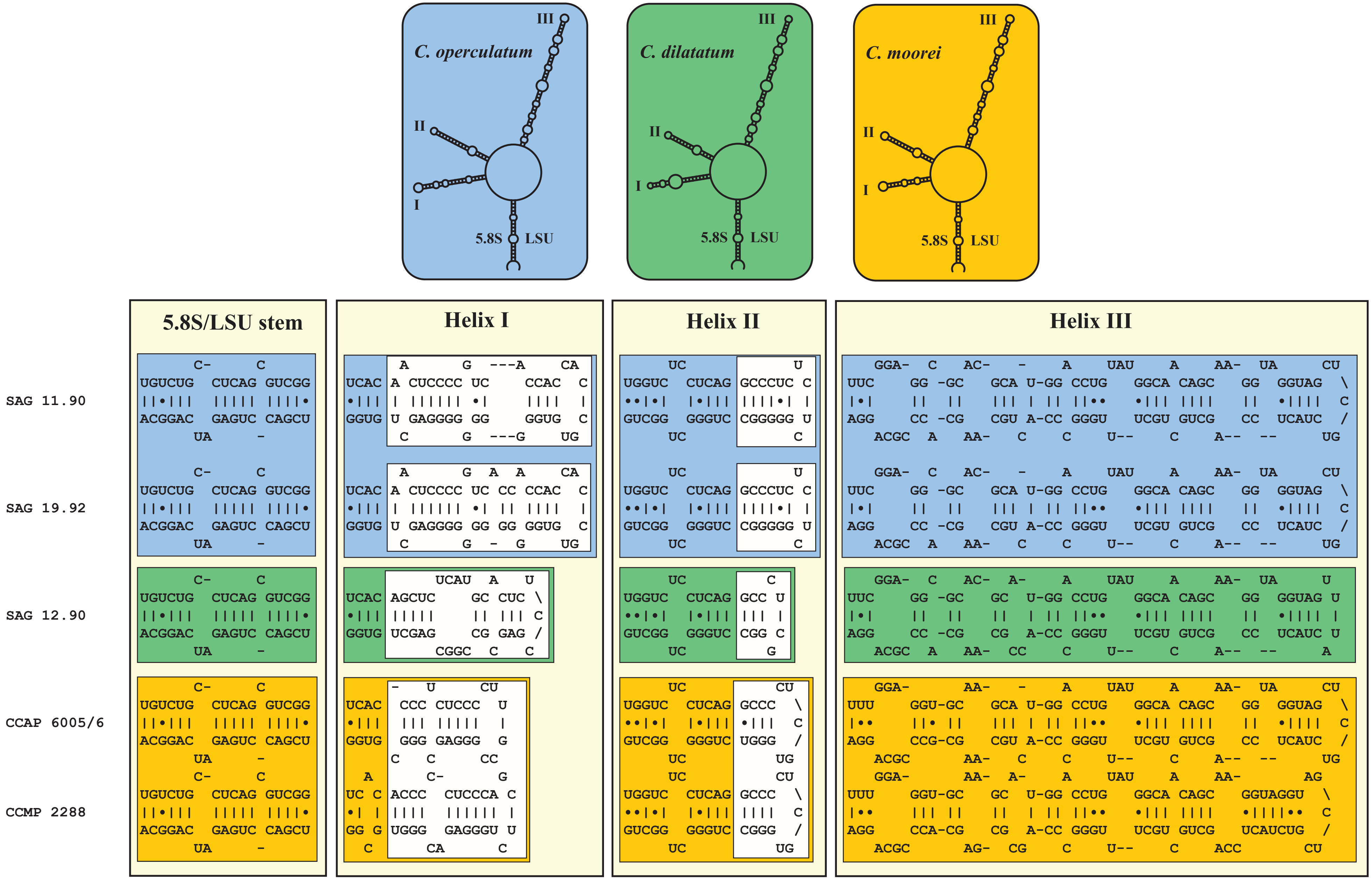
C. operculatum
C. dilatatum
C. moorei
III
II
I
5.8S
LSU
5.8S/LSU stem
Helix I
Helix II
Helix III
SAG 11.90
SAG 19.92
SAG 12.90
CCAP 6005/6
CCMP 2288
