# Supplementary material for: Molecular Phylogeny of Unicellular Marine Coccoid Green Algae Revealed New Insights into the Systematics of the Ulvophyceae (Chlorophyta)
Source: Microorganisms. 2021 Jul 26;9(8):1586. doi: 10.3390/microorganisms9081586 (PMC8401757; doi:10.3390/microorganisms9081586)
Supplement: Supplementary file 1 [file microorganisms-09-01586-s001.zip › Figure_S4.pdf]

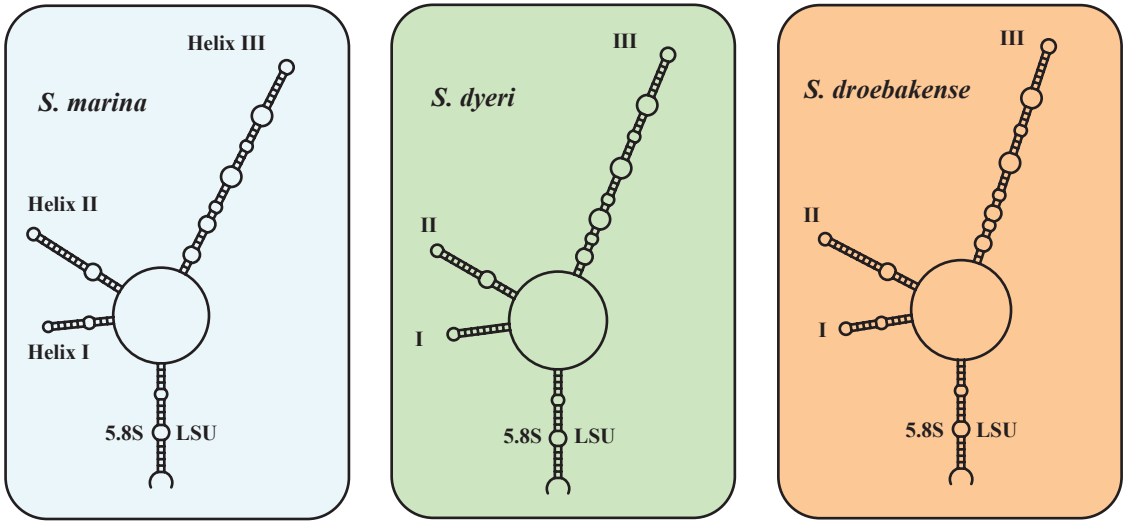

5.8S/LSU stem

```
                C-      C
UTEX 1445   UGUCUG   CUCAG GUCGG
            ||•|||   ||||| |||||•
            ACGGAC   GAGUC CAGCU
                UA      -

                C-      C
CCMP 257    UGUCUG   CUCAG GUCGG
            ||•|||   ||||| |||||•
            ACGGAC   GAGUC CAGCU
                UA      -

                C-      C
CCMP 258    UGUCUG   CUCAG GUCGG
CCMP 438    ||•|||   ||||| |||||•
            ACGGAC   GAGUC CAGCU
                UA      -
```

Helix I

```
                C       A
UTEX 1445   UCAC  CUCCAA \
            •|||   |•|||| C
            GGUG   GGGGUU /
                A       A

                      U
CCMP 257    UCAC  CCCCC C
            •|||  ||||| |
            GGUG  GGGGG A
                      C

                C     C
CCMP 258    UCAC  C CCUAC U
CCMP 438    •|||  | ||||| |
            GGUG  G GGAUG A
                   A    G
```

Helix II

```
                UU          C
UTEX 1445   UGGUC  CCCAG CGGUG U
            ••|•|   ||||| |||•| |
            GUCGG  GGGUC GCCGC U
                UC          G

                UC          C
CCMP 257    UGGUC  CUCAG CCG U
            ••|•|   |•||| ||| |
            GUCGG  GGGUC GGC C
                UU          G

                UC          C
CCMP 258    UGGUC  CUCAG CUGCG U
CCMP 438    ••|•|   |•||| ||•|| |
            GUCGG  GGGUC GAUGC U
                UU          G
```

Helix III

```
                AC     --UU  A    UAU     A    AA-         AG
UTEX 1445   GGU   GCGC--   GG CCUG    GGCA CAGC    GGUAGGU   \
            |||   ||||--   |• ||••    •||| ||||    •||||••   C
            CCA   CGCG--   CU GGGU    UCGU GUCG    UCAUCUG   /
                GU     --UU  C    U--     A    ACC         CU

                --  G  CUU  A    UAU     A    AA-         AG
CCMP 257    GGU   GC CG    GG CCUG    GGCA CAGC    GGUAGGU   \
            |||   || ||    || ||••    •||| ||||    •||||••   C
            CCA   CG GC    CC GGGU    UCGU GUCG    UCAUCUG   /
                AA  G  -U-  A    U--     A    ACC         UU

                GC  G --UU  A    UAU     A    AA-         AG
CCMP 258    GGU   GC C--   GG CCUG    GGCA CAGC    GGUAGGU   \
CCMP 438    |||   •| |--   || ||••    •||| ||||    •||||••   C
            CCA   UG G--   CC GGGU    UCGU GUCG    UCAUCUG   /
                AA  A --UU  A    U--     A    ACC         UU
```
