# Supplementary material for: Molecular Phylogeny of Unicellular Marine Coccoid Green Algae Revealed New Insights into the Systematics of the Ulvophyceae (Chlorophyta)
Source: Microorganisms. 2021 Jul 26;9(8):1586. doi: 10.3390/microorganisms9081586 (PMC8401757; doi:10.3390/microorganisms9081586)
Supplement: Supplementary file 1 [file microorganisms-09-01586-s001.zip › Table_S1.pdf]

species		Haplotype	Habitat	continent
Desmochloris halophila	CCAP 6006/1	D1a	marine	North America (USA, MA)
	CCAP 6006/4	D1a	soil	South America
	KT860927	D1a	marine	Europe, North Sea
	MH703754	D1b (*)	soil	West Europe
	SAG 2397	D1c	freshwater	West Europe
Desmochloris edaphica	CCAP 6006/5	D2	soil	East Europe
	CCAP 6006/6	D2	soil	South America
	MT968399	D2	soil, saline	West Europe
	MH102328	D2	soil	East Europe
	KY086483	D2	soil	East Europe
Desmochloris mollenhaueri	CCAP 6006/2	D3a	soil	Africa
	CCAP 6006/3	D3a	soil	Africa
	CCAP 6006/7	D3a	soil	Africa
	CCAP 6006/8	D3a	soil	South America
	CCAP 6006/9	D3a	soil	South America
	KF791549	D3b (*)	soil saline	Asia China
Chlorocystis cohnii	SAG 9.90	C1	marine	West Europe
	SCCAP K-0421	C1	marine, endophyte	North America, Greenland
Chlorocystis dangeardii	SAG 8.86	C2	marine	West Europe
	CCAP 211/25	C2	brackish	West Europe
	CCAP 233/1	C2	marine	West Europe
Chlorocystis john-westii	CCAP 6005/4	C3	marine, endophyte	South America
	CCAP 6005/5	C3	marine, endophyte	Australia
	CCAP 6005/10	C3	marine, endophyte	South America
	CCAP 6005/11	C3	marine, endophyte	Australia
	CCAP 6005/12	C3	marine, endophyte	Australia
	UTEX 2846	C3	marine, endophyte	South America
	MK541803	C3	marine	Africa
	AB058345	C3	marine	Asia
Chlorocystis operculatum/ dilatatum	SAG 19.92	C4a	marine	West Europe
	CCMP 435	C4a	marine	West Europe
	SAG 11.90	C4a	marine	West Europe
	SAG 12.90	C4a	marine	West Europe
	MG647626	C4b	marine	Asia
Chlorocystis moorei	CCAP 6005/6	C5	marine	West Europe
	CCMP 2288	C5	marine	North America (USA, Washington)
	MT968404	C5	soil, saline	West Europe
	MT968406	C5	soil, saline	West Europe
	MT968405	C5	soil, saline	West Europe
	MT968403	C5	soil, saline	West Europe
	MT968402	C5	soil, saline	West Europe
	MT968401	C5	soil, saline	West Europe
	DQ821520	C5	marine	North America (USA, California)
Sykidion marinum	UTEX 1445	S1	unknown	unknown
Sykidion dyeri	CCMP 257	S2	marine	North America (USA, CT)
	MK457458	S2	marine	South America (Brasil)
Sykidion droebakense	CCMP 258	S3a	marine	North America (British Columbia)
	CCMP 438	S3a	marine	Antarctica
	LC505539	S3b	marine	Asia

(*) These entries contains sequencing mistakes as demonstrated in Figure 5.
