# Supplementary figures and images for: Molecular Phylogeny of Unicellular Marine Coccoid Green Algae Revealed New Insights into the Systematics of the Ulvophyceae (Chlorophyta)
Source: Microorganisms. 2021 Jul 26;9(8):1586. doi: 10.3390/microorganisms9081586 (PMC8401757; doi:10.3390/microorganisms9081586)

**SSU rRNA secondary structure model  
of *Chlorocystis cohnii*  
strain SAG 9.90  
accession number: MW714132**

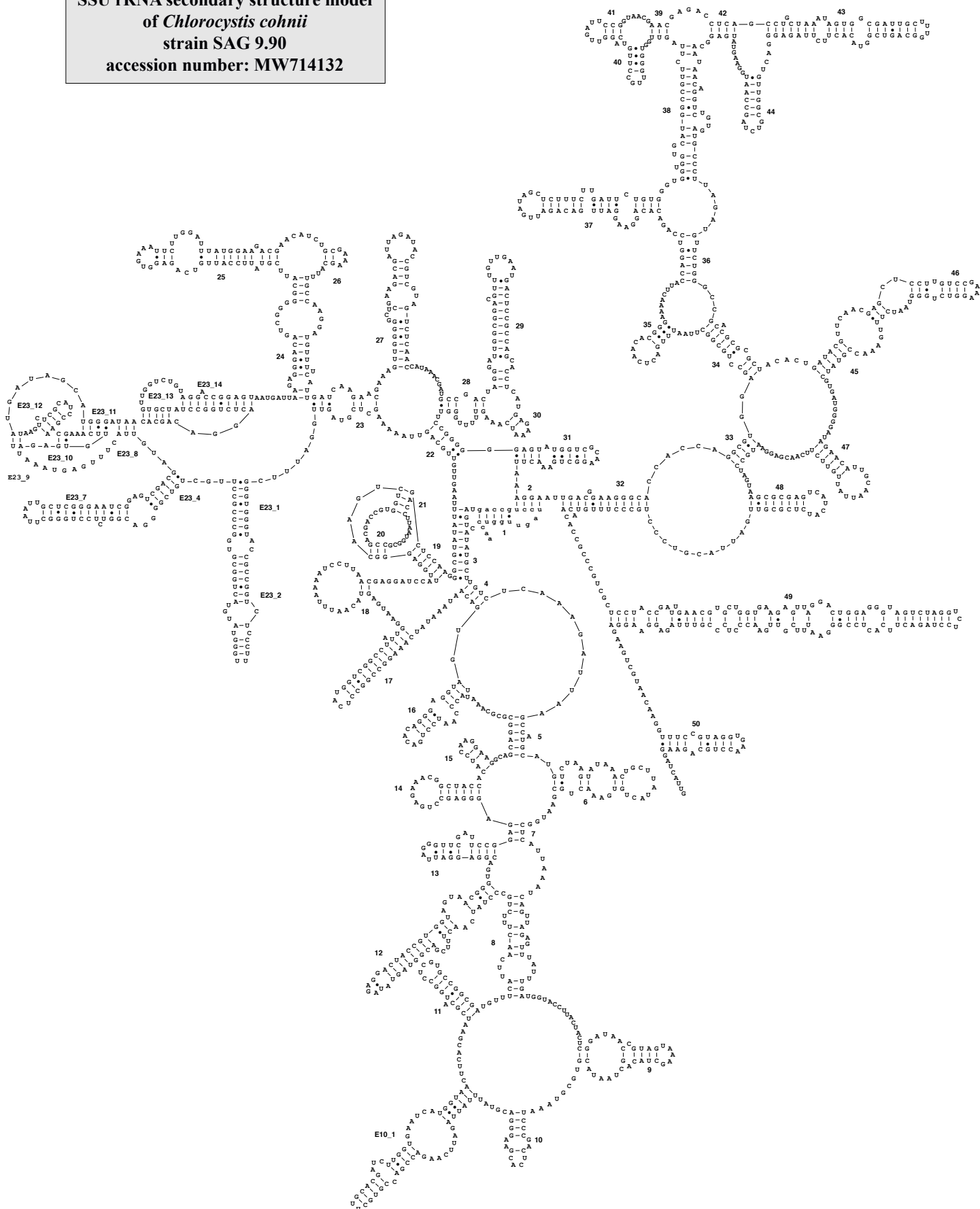

Supplement: Supplementary file 1 [file microorganisms-09-01586-s001.zip › Figure_S1.pdf]
